# Gastruloid-derived primordial germ cell-like cells develop dynamically within integrated tissues

**DOI:** 10.1242/dev.201790

**Published:** 2023-09-11

**Authors:** Christopher B. Cooke, Christopher Barrington, Peter Baillie-Benson, Jennifer Nichols, Naomi Moris

**Affiliations:** ^1^ The Francis Crick Institute, 1 Midland Road, London NW1 1AT, UK; ^2^ Department of Genetics, University of Cambridge, Cambridge CB2 3EH, UK; ^3^ Abcam, Discovery Drive, Cambridge Biomedical Campus, Cambridge CB2 0AX, UK; ^4^ Wellcome Trust – MRC Stem Cell Institute, University of Cambridge, Jeffrey Cheah Biomedical Centre, Puddicombe Way, Cambridge CB2 0AW, UK; ^5^ Department of Physiology, Development and Neuroscience, University of Cambridge, Tennis Court Road, Cambridge CB2 3EG, UK; ^6^ Centre for Trophoblast Research, University of Cambridge, Cambridge, UK

**Keywords:** Primordial germ cell, Gastruloid, Embryo, Stem cell, Cell interactions

## Abstract

Primordial germ cells (PGCs) are the early embryonic precursors of gametes – sperm and egg cells. PGC-like cells (PGCLCs) can currently be derived *in vitro* from pluripotent cells exposed to signalling cocktails and aggregated into large embryonic bodies, but these do not recapitulate the native embryonic environment during PGC formation. Here, we show that mouse gastruloids, a three-dimensional *in vitro* model of gastrulation, contain a population of gastruloid-derived PGCLCs (Gld-PGCLCs) that resemble early PGCs *in vivo*. Importantly, the conserved organisation of mouse gastruloids leads to coordinated spatial and temporal localisation of Gld-PGCLCs relative to surrounding somatic cells, even in the absence of specific exogenous PGC-specific signalling or extra-embryonic tissues. In gastruloids, self-organised interactions between cells and tissues, including the endodermal epithelium, enables the specification and subsequent maturation of a pool of Gld-PGCLCs. As such, mouse gastruloids represent a new source of PGCLCs *in vitro* and, owing to their inherent co-development, serve as a novel model to study the dynamics of PGC development within integrated tissue environments.

## Teaser

**Highlighted Article:** Mouse gastruloids, 3D *in vitro* models of gastrulation, contain a population of primordial germ cell (PGC)-like cells that resemble early PGCs *in vivo* and are closely associated with neighbouring tissues during their development.

## INTRODUCTION

The specification of mouse primordial germ cells (PGCs) occurs at the gastrulation stage epiblast at about embryonic day (E) 7.25, when competent cells begin to co-express *Stella* (also known as *Dppa3*) and *Blimp1* (*Prdm1*) and become lineage-restricted to a germ cell fate (Lawson and Hage, 1994; Saitou et al., 2002; Sato et al., 2002; Ohinata et al., 2005) by repression of somatic genes and the activation of the PGC-specific program (Vincent et al., 2005; Yabuta et al., 2006). This specification occurs at the proximal posterior of the epiblast and is thought to be dependent on signals from the extra-embryonic ectoderm and visceral endoderm, including BMP (Saitou et al., 2002) and Wnt signalling (Ohinata et al., 2009), as embryos mutant for *Bmp4* or one of its receptors, *Alk2* (*Acvr1*), have reduced numbers of PGCs (Lawson et al., 1999; de Sousa Lopes et al., 2004). After specification, PGCs are incorporated into the developing hindgut and move anteriorly through this tissue before then migrating through the dorsal mesentery towards the genital ridge (Gomperts et al., 1994; Molyneaux and Wylie, 2004), the precursors of the gonads. Here, the germ cells colonise the prospective gonadal niche in the form of small cell clusters (Gomperts et al., 1994) and continue to mature in terms of their transcriptional and, particularly, their epigenetic signature. At approximately E12.5 (Gill et al., 2011; Nicholls et al., 2019), sexual determination occurs, and initiates further sex-specific maturation that ultimately generates spermatozoa in males and oocytes in females. Their time-course is therefore highly dynamic and occurs through close association with several different tissues and cell types of the developing embryo (Cooke and Moris, 2021).

Currently, pluripotent stem cell-based PGC-like cell (PGCLC) *in vitro* models (Hayashi et al., 2011, 2018) have been used to explore the regulatory mechanisms of early specification and maturation (for example, Aramaki et al., 2013; Nakaki et al., 2013) and even to generate mature germ cells through gametogenesis (Hayashi et al., 2011, 2012; Hayashi and Saitou, 2013; Hikabe et al., 2016; Ishikura et al., 2016; Morohaku et al., 2016; Yoshino et al., 2021). These models are typically derived from epiblast-like cells, which are subsequently arranged as embryoid bodies (EBs), and they build on earlier work that observed spontaneous PGCLC differentiation in EBs (Hübner et al., 2003; Toyooka et al., 2003; Geijsen et al., 2004), but with the addition of PGC-specific factors to strongly bias towards a PGCLC fate. Yet, despite being an efficient protocol, these EB-derived PGCLCs are formed within largely disorganised aggregates of cells that lack the spatially organised, supportive neighbouring cell types found in the embryo, and have limited epigenetic remodelling towards mature germ cells (Ge et al., 2015; Ramakrishna et al., 2022). In addition, further maturation of PGCLCs beyond the gonadal colonisation stage *in vitro* currently requires complete dissociation of EBs and reaggregation with gonadal cell populations (Hayashi et al., 2012; Hayashi and Saitou, 2013; Hikabe et al., 2016; Ishikura et al., 2021), which necessarily results in loss of any endogenous spatial colocalisation or organisation and precludes any study of the gradual developmental dynamics of PGCLCs during this maturation time window. Therefore, although EB-based methods provide a readily available source of *in vitro* PGCLCs, these methods are unable to reveal the complexities of PGC specification or their interaction with the rest of the embryonic body plan in a developmentally faithful manner.

Recently, mouse gastruloids, three-dimensional mouse embryonic stem cell (mESC)-derived aggregates, have been described and characterised to undergo gastrulation-like gene expression progression, multilineage differentiation, axial polarisation and morphological extension (van den Brink et al., 2014; Beccari et al., 2018). Single-cell analysis showed that these gastruloids include many cell types found in the early mouse embryo, including a population of presumptive PGCLCs (van den Brink et al., 2020). Others have also shown that small populations of *Sox2*^+^/Stella^+^ cells (Veenvliet et al., 2020) and DPPA4^+^ cells (Vianello and Lutolf, 2020 preprint) exist along the anteroposterior length of gastruloid-like structures. Here, we report the further characterisation of these gastruloid-derived PGCLCs (Gld-PGCLCs), including their dynamic spatiotemporal localisation and association within integrated tissue environments. Importantly, we show that Gld-PGCLCs display characteristics that are akin to *in vivo* PGCs and that they recapitulate features of early PGC migration and maturation, reaching stages equivalent to ∼E14.5, while relying mainly on endogenous inductive signals from within the self-organised gastruloid.

## RESULTS

### Identification of mouse Gld-PGCLCs

**Fig. 1.**
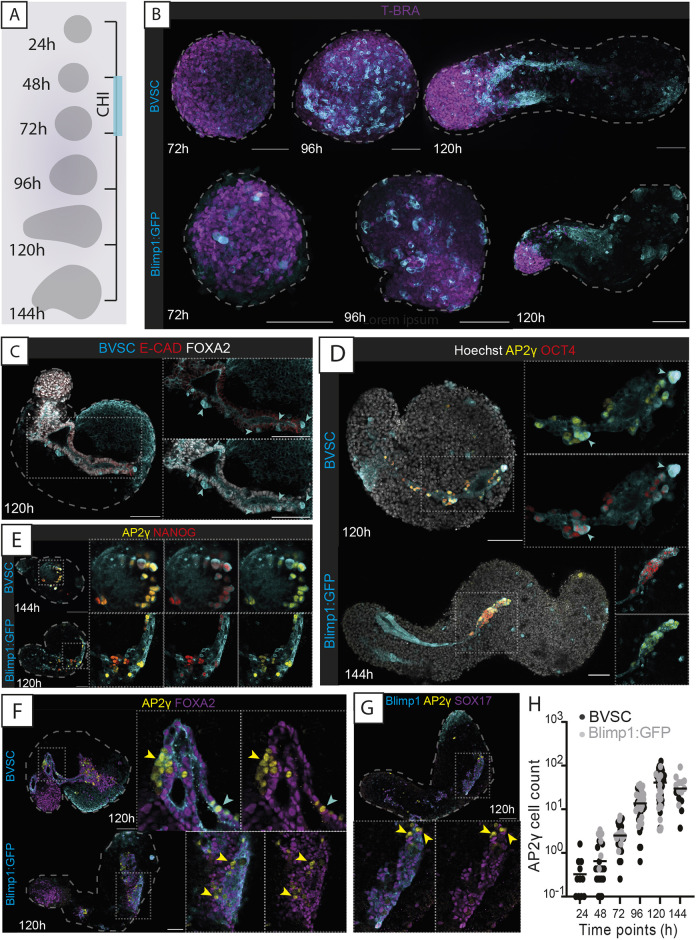
**Characterising gastruloid-derived PGCLCs.** (A) Schematic of gastruloid protocol and morphological changes from 24 to 144 h. CHI, CHIR-99021. (B) Maximum-projection images of gastruloids from BVSC and Blimp1:GFP reporter lines. In BVSC gastruloids, Blimp1:mVenus is membrane-targeted, whereas Stella:eCFP is found throughout the cell. (C) *Z*-section images of Blimp1-mVenus^+^ endodermal tracts. (D) Expression of AP2γ and OCT4 in gastruloids. (E) Expression of AP2γ and NANOG in gastruloids. (F,G) AP2γ-expressing cells do not co-express FOXA2 (F) or SOX17 (G). (H) Cell counts of AP2γ-expressing cells from both Blimp1:GFP and BVSC gastruloids. Black bars represent the mean value at each time point. In B-E: cyan arrowheads, Stella^+^ cells; yellow arrowheads, AP2γ^+^ cells; insets, higher-magnification images; dashed lines, morphological gastruloid outline from Hoechst staining; dotted lines, magnification regions. Images are representative of 4-32 gastruloids per panel. Scale bars: 100 µm.

The transcriptional expression of both *Blimp1* and *Stella* is associated with PGCs in the mouse embryo (Ohinata et al., 2005; Hayashi et al., 2007). We therefore generated mouse gastruloids using the Blimp1:eGFP (herein, Blimp1:GFP) (Ohinata et al., 2005) and Blimp1:mVenus Stella:eCFP (BVSC) (Borggrefe and Oswald, 2009) mESCs, which have previously been used as markers of PGCLC state *in vitro* (Hayashi and Surani, 2009). Aggregates made from BVSC and Blimp1:GFP cells broke symmetry at approximately 96 hours after aggregation (hereafter ‘h’), leading to elongated structures with polarised expression of the mesodermal marker brachyury (T-BRA, encoded by *T*) and CDX2 at 120 h (Fig. 1A,B; Fig. S1A), which are comparable with gastruloids generated from E14tg2A cells (van den Brink et al., 2014; Turner et al., 2017) routinely cultured in 2iLIF (see Materials and Methods) (Anlas et al., 2021; Cermola et al., 2021).

We therefore examined the dynamic expression of the PGC-associated gene reporters in these gastruloids. *Blimp1* is expressed in the endoderm of the mouse embryo (Vincent et al., 2005), and the coalescence of endodermal domains into a tube structure in mouse gastruloids has been previously described (Beccari et al., 2018; Vianello and Lutolf, 2020 preprint; Hashmi et al., 2022). In our gastruloids, Blimp1 expression was observed initially in a salt-and-pepper manner across spherical gastruloids at 72 h, which then tended to coalesce into domains or clusters of expression in ovoid-shaped gastruloids at 96 h (Fig. 1B). As mouse gastruloids underwent elongation, the domain of Blimp1 expression became even more spatially defined, and contiguous tracts of *Blimp1*-expressing cells running along the anteroposterior axis were routinely formed by 120 h [apparent in 78.3% BVSC (*n*=60) and 60.8% Blimp1:GFP (*n*=74) gastruloids; Fig. 1B]. These Blimp1*^+^* tracts also expressed FOXA2, SOX17, E-cadherin (CDH1) and EpCAM (Fig. S1B-F), suggesting a definitive endoderm identity. They were internally located and typically formed closed, tube-like structures (Fig. S1G).

Although the majority of Blimp1-expressing cells in gastruloids therefore likely represent a definitive endodermal population, we observed several Blimp1- and Stella-co-expressing cells that were interspersed within or adjacent to the endoderm tubes in BVSC gastruloids (Fig. 1C; Fig. S1F,G). We reasoned that these were likely to be PGCLCs. Indeed, the PGC marker AP2γ (encoded by *Tfap2c*) was found to be co-expressed with the pluripotency factor OCT4 (also known as POU5F1) and NANOG in a high proportion of these cells (Fig. 1D,E; Fig. S1H), which did not express the endodermal markers FOXA2 or SOX17 (Fig. 1F,G). Although Stella expression was consistently observed in mouse gastruloids, not all Stella*^+^* cells were positive for both OCT4 and AP2γ and often co-expressed only one of these markers (Fig. S1H-J), suggesting that there might be heterogeneity of Stella-expressing cells in Gld-PGCLCs, perhaps related to the temporal range of states observed. Therefore, we also used platelet and endothelial cell adhesion molecule 1 (PECAM1) expression, which is known to be expressed in PGCs in the mouse embryo (as well as in pluripotent and endothelial cells) (Baldwin et al., 1994; Robson et al., 2001; Wakayama et al., 2003; Li et al., 2005). In 120 h gastruloids, PECAM1 was co-expressed in the majority of AP2γ- (96.65%), OCT4- (95.83%) and Stella*-* (98.3%) positive cells in BVSC gastruloids, and when we examined AP2γ expression alongside PECAM1, we observed double-positive cells as early as 24 h, which then co-expressed Stella from 72 h (Fig. S2A-C), suggesting that PECAM1 marked the broadest population of Gld-PGCLCs across the time course. We therefore decided to use both AP2γ and PECAM1, in combination with the endogenous reporters of BVSC or Blimp1:GFP cell lines, as general markers of Gld-PGCLCs.

Gastruloids displayed a consistent and progressive increase in the number of Gld-PGCLCs through the gastruloid timeline from 24 h to 120 h (Fig. 1H). This began as an average (±s.d.) of 2.42±2.15 cells per gastruloid (8.3% of gastruloids had no AP2γ-expressing cells), which increased to 4.07±4.16 cells at 48 h (20% of gastruloids without AP2γ^+^ cells) and continued to increase to reach an average of 90.72±49.11 cells per gastruloid by 120 h (0% of gastruloids had no AP2γ expression, *n*=57). At 144 h, the average number of Gld-PGCLCs slightly decreased (71.93±37.91 cells; Fig. 1H), which mirrored a general decrease in the average size of 144 h gastruloids (Table S1). Likewise, by flow cytometric analysis, a population that was doubly positive for Stella:eCFP and PECAM1 was observed to increase in frequency during BVSC gastruloid development (Fig. S2C). These estimates of absolute Gld-PGCLC numbers are roughly consistent with the equivalent *in vivo* PGC numbers, with approximately ∼100 PGCs found in the E8.5 mouse embryo (Ginsburg et al., 1990; Seki et al., 2007), which represents an equivalent stage to 120 h gastruloids (Beccari et al., 2018; van den Brink et al., 2020) and an average doubling time approximating 16.12 h (Fig. 1H), matching the 16 h estimated for mouse PGCs in the embryo (Tam and Snow, 1981).

### Dynamic localisation of Gld-PGCLCs

**Fig. 2.**
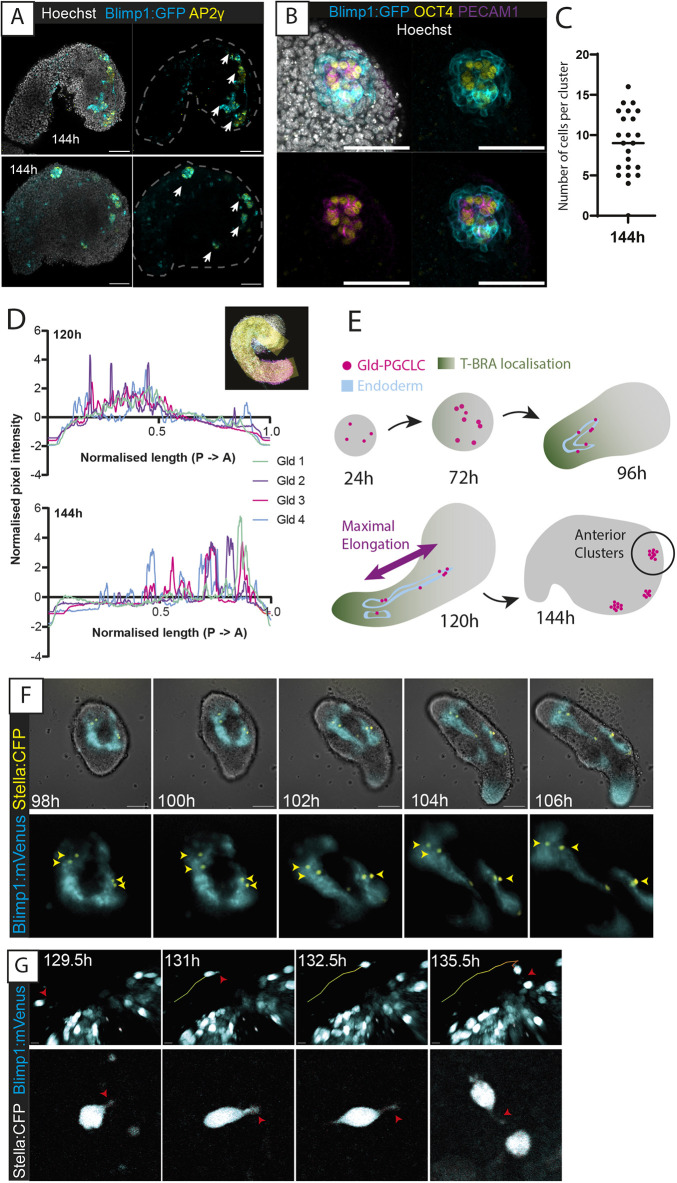
**Anterior localisation and movement of Gld-PGCLCs.** (A) Anterior-localised clusters of AP2γ^+^ cells at 144 h. White arrows indicate locations of discrete clusters. Scale bars: 100 µm. (B) High-magnification *z*-slice of an OCT4^+^ and PECAM1^+^ cluster at 144 h. Scale bars: 100 µm. (C) Quantification of the number of cells in each cluster at 144 h, as determined by co-expression of at least two of Blimp1, AP2γ, PECAM1 or DAZL. *n*=7 gastruloids. The black bar indicates the median average. (D) Anteroposterior localisation of AP2γ^+^ cells along the gastruloid length (see Materials and Methods). ‘Gld’ indicates individual gastruloid replicates. Inset, representation of a 120-pixel-width line spanning the anteroposterior axis of the *z*-stack maximum projection of the gastruloid. ‘A’, anterior; ‘P’, posterior. (E) Schematic representation of Gld-PGCLC localisation within gastruloids across their time course. (F) Widefield time-lapse imaging of a BVSC gastruloid from 98 to 106 h. Top, whole-gastruloid image; bottom, zoom-in of fluorescent reporter domain. Yellow arrowheads, Stella^+^ cells. Scale bars: 100 µm (top row). (G) Multiphoton time-lapse images of a BVSC gastruloid from 129.5 to 135.5 h with cell tracking (plotted line). Red arrowheads, cell morphological features associated with active migration. Scale bars: 10 µm (top row). Images are representative of 9-11 gastruloids per panel.

We were particularly interested to note the spatial localisation of the Gld-PGCLCs relative to the endodermal tract, given the role of PGC migration along the endoderm *in vivo* (Anderson et al., 2000; Molyneaux et al., 2001). We noted that at 120 h, Gld-PGCLCs were often interspersed throughout the endodermal tract along the anteroposterior axis, but by 144 h, the majority were localised within small clusters of cells at the anterior edge of gastruloids (Fig. 2A,B). These each contained an average of nine cells expressing two or more PGC-associated proteins, and each gastruloid had on average 3.3 clusters (*n*=7) (Fig. 2C; Table S2), similar to PGCs colonising mouse gonads at E10.5 (Pepling and Spradling, 1998). As Gld-PGCLCs seemed to shift towards the anterior end of the gastruloids relative to the length of the gastruloid [average (±s.d.) location at 75.9±9.95% of the gastruloid length starting from the posterior at 144 h, *n*=15; Fig. 2D], we reasoned that they might be moving relative to the axis of maximal elongation of the gastruloid from posterior to anterior (Fig. 2E).

Indeed, we observed evidence of Gld-PGCLC movement throughout their development in gastruloids. Some of this movement appeared to be due to overall morphological changes associated with gastruloid elongation and might, therefore, represent a passive relative movement of the Gld-PGCLCs. For instance, Gld-PGCLCs (Stella:eCFP-expressing) were often already intermingled with endodermal cells (Blimp1:Venus-expressing) prior to elongation at 96 h (*n*=29/39 gastruloids), and later became distributed throughout the endodermal tracts, concurrent with gastruloid elongation (Fig. 2F; Movie 1). As E-cadherin and EpCAM were expressed in both Gld-PGCLCs and endodermal cells at 96 h (Fig. S3A,B), it is possible that this co-expression between tissues could potentially mediate the observed close association between the tissues, as has been suggested in the mouse embryo (Bendel-Stenzel et al., 2000; Di Carlo and De Felici, 2000), although further experiments would be required to test this hypothesis.

In addition, it is likely that Gld-PGCLCs are also capable of active movement as well as passive relative movement. Using multiphoton microscopy, we observed several instances of Stella-positive cells displaying seemingly motile behaviour relative to the gastruloid structure, as well as morphological changes associated with migration, including cellular protrusions that appear filopodia-like, and interactions with other Stella^+^ cells (Fig. 2G; Movie 2). However, such movement was not always strictly posterior-to-anterior, and thus the observed shift in the relative location of Gld-PGCLCs is likely to be due to both active and passive movement of cells.

**Fig. 3.**
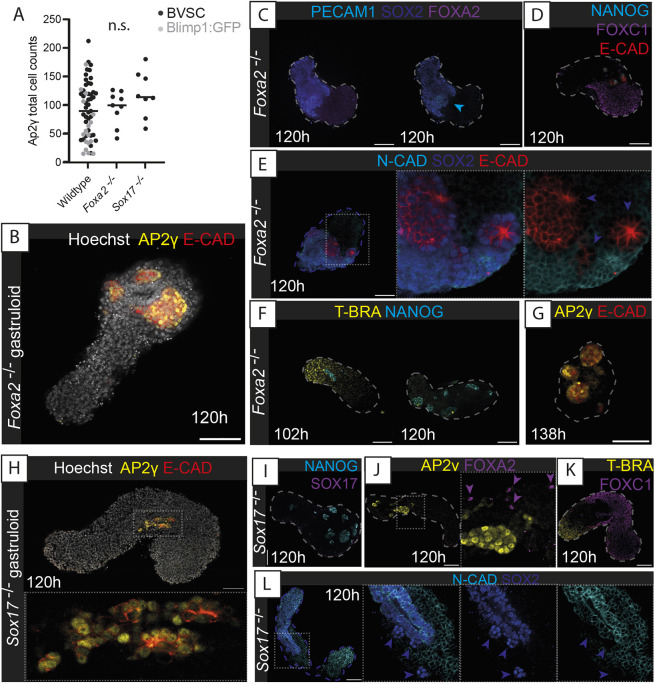
**Knockout of gastruloid endodermal tissue leads to aberrant Gld-PGCLC localisation but maintains mesodermal and ectodermal populations.** (A) Quantification of AP2γ^+^ cell counts in Blimp1:GFP and BVSC gastruloids (wildtype), alongside *Foxa2*^−/−^ and *Sox17*^−/−^ gastruloids. Black bars indicate the median average; n.s., no significant difference. (B) AP2γ^+^ cells localise into large clusters in *Foxa2*^−/−^ gastruloids and show no E-cadherin (E-CAD-positive) endodermal tracts (AP2γ negative). (C) Confirmation of lack of FOXA2 expression in *Foxa2*^−/−^ gastruloids. Blue arrowhead indicates a population of SOX2^+^, PECAM1^+^ PGCLCs. (D) Maintenance of FOXC1 mesoderm in *Foxa2*^−/−^ gastruloids at 120 h. (E) Neural ectodermal cell types present in *Foxa2*^−/−^ gastruloids as evidenced by N-Cadherin (N-CAD or CDH2) and SOX2 expression. (F) Mesodermal T-BRA expression in *Foxa2*^−/−^ gastruloids was seen at 102 h but not at 120 h. (G) Later-stage putative Gld-PGCLC in 138 h *Foxa2*^−/−^ gastruloids. (H) AP2γ^+^ cells localise into large clusters in *Sox17*^−/−^ gastruloids and show no E-cadherin (E-CAD)-positive endodermal tracts (AP2γ negative). (I) Confirmation of lack of SOX17^−/−^ expression detected in *Sox17*^−/−^ gastruloids. (J) Presence of several scattered FOXA2^+^ cells (purple arrowheads) in *Sox17*^−/−^ gastruloids. (K) Maintenance of FOXC1 mesoderm in *Sox17*^−/−^ gastruloids at 120 h. (L) Neural ectodermal cell types present in *Sox17*^−/−^ gastruloids as evidenced by N-Cadherin (N-CAD) and SOX2 expression. Violet arrowheads, SOX2^+^, N-CAD^−^ cells likely to be Gld-PGCLCs. In E,H,J,L, insets show higher-magnification images. Dashed lines, morphological gastruloid outline from Hoechst staining; dotted lines, magnification regions. Images are representative of 4-13 gastruloids per panel. Scale bars: 100 µm.

Given the apparent role of the endodermal epithelium to coordinate the relative localisation of Gld-PGCLCs to the anterior end of gastruloids, we wanted to investigate the necessity of this endodermal population for Gld-PGCLC localisation. In mice, *Sox17*-null embryos specify PGCs, but they cannot enter the gut endoderm and are stalled at the hindgut entrance (Hara et al., 2009). We therefore generated mouse gastruloids from *Sox17*^−/−^ (see Materials and Methods) or *Foxa2*^−/−^ (Cernilogar et al., 2019) mESCs (Fig. 3A-L). In both cases, the mutant gastruloids still contained the mesoderm and the ectoderm and underwent axial elongation, but the endodermal population was absent and no epithelial tract was observed. The Gld-PGCLC population was observed at absolute cell numbers equivalent to those of wildtype gastruloids (Fig. 3A), but, importantly, they were localised in large clusters at 120 h rather than being dispersed throughout the length of the gastruloid (Fig. 3B,H). This observation strongly supports the notion that the presence of the endodermal tract in gastruloids facilitates the spatially organised movement of Gld-PGCLCs, closely resembling observations in the mouse embryo (Hara et al., 2009).

### Maturation of Gld-PGCLCs

**Fig. 4.**
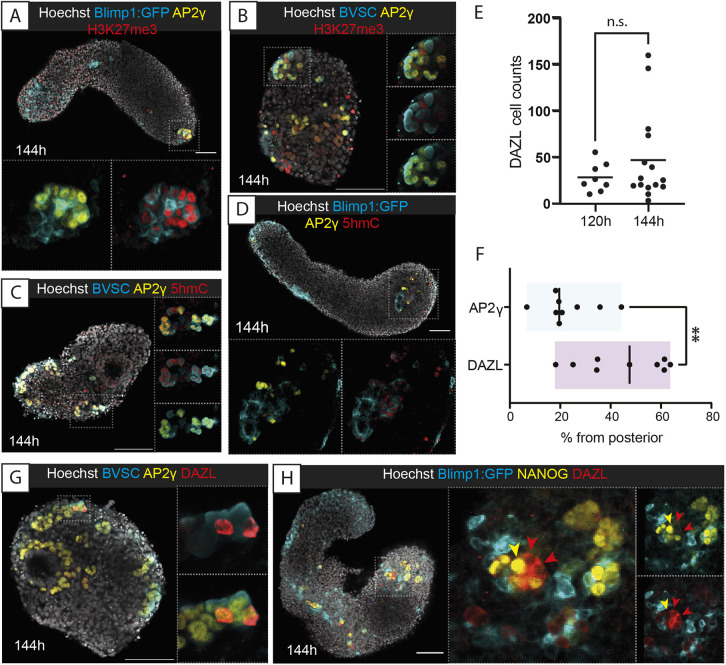
**Maturation of Gld-PGCLCs and epigenetic and protein expression changes associated with germ cell determination.** (A,B) Histone H4 trimethylation of K27 (H3K27me3) localization in Gld-PGCLCs in Blimp1:GFP (A) and BVSC (B) gastruloids at 144 h. In BVSC gastruloids, Blimp1:mVenus is membrane-targeted, whereas Stella:eCFP is found throughout the cell. (C,D) 5-hydroxymethylcytosine (5hmC) localization in Gld-PGCLCs in Blimp1:GFP (C) and BVSC (D) gastruloids at 144 h. (E) Quantification of DAZL-expressing cells in BVSC and Blimp1:GFP gastruloids. Black line represents the mean cell count. n.s., no significant differences. (F) Quantification of Gld-PGCLC localisation along the anteroposterior axis, using the posterior-most detected expression from each gastruloid as a percentage of total length (see Materials and Methods for details). Black line represents the median value. **P<0.01 (unpaired, two-tailed *t*-test with Welch's correction). (G,H) DAZL expression in 144 h BVSC (G) and Blimp1:GFP (H) gastruloids. Yellow arrowhead, NANOG^+^ DAZL^−^ cell; red arrowheads, NANOG^−^ DAZL^+^ cells. Insets, higher magnification images; dotted lines, magnification region. Images are representative of 3-11 gastruloids per panel. Scale bars: 100 µm.

The morphological clustering of Gld-PGCLCs in the anterior of 144 h gastruloids was highly reminiscent of gonadal germ cell clusters found in the mouse embryo at E11.5 (Gomperts et al., 1994; Pepling and Spradling, 1998). We therefore wondered whether these anterior-localised Gld-PGCLCs were undergoing further maturation, particularly in the form of epigenetic remodelling. Indeed, we observed that the histone modification H3K27me3, which has been shown to be associated with PGC maturation to a germ cell fate (Seki et al., 2005; Hajkova et al., 2008), was co-localised with AP2γ at 144 h (35% co-expression; Fig. 4A,B; Table S3). Similarly, the DNA modification mark 5-hydroxymethylcytosine (5hmC) was also colocalised with Gld-PGCLCs in anterior clusters of cells in 144 h gastruloids (45% co-expression; Fig. 4C,D; Table S3), another hallmark of PGC maturation (Hajkova et al., 2002; de Napoles et al., 2004). As DNA demethylation is required to de-repress the promoter of the germ cell gene *Dazl*, which itself is required to facilitate the maturation of germ cells towards sex-specific stages in a process called ‘licensing’ (Gill et al., 2011), we examined the expression of DAZL in gastruloids. Surprisingly, we observed clear DAZL protein expression in Gld-PGCLCs at 120 h (mean±s.d.=28±15.46 cells per gastruloid, *n*=8), which stayed consistent in 144 h gastruloids (46.6±47.93 cells per gastruloid, *n*=15; Fig. 4E) and was localised particularly in anterior clusters (Fig. 4F). Furthermore, DAZL was co-expressed with AP2γ (21% co-expression; Fig. 4G; Table S3) and we generally found DAZL expression in cells that had lower levels of NANOG expression (Fig. 4H), potentially relating to its role in downregulating pluripotency factors during germ cell maturation (Chen et al., 2014). As such, it seems that the Gld-PGCLCs begin to undergo a maturation process that, to some extent, mirrors the post-migratory/gonadal-stage development of PGCs *in vivo* and that might be directly mediated by their local environment.

We hypothesised that local signalling or niche properties of surrounding cells in the anterior region of the gastruloid could be supporting these cell clusters. Indeed, we frequently observed high expression of GATA4 in several cells near the Gld-PGCLC clusters (Fig. S4A-C). In support of this observation, closer examination of extant spatial transcriptomics datasets from 120 h mouse gastruloids (van den Brink et al., 2020) showed an anterior localisation of *Gata4*, an early marker of the developing bipotent gonad (Hu et al., 2013), and *Cxcl12* (also known as *Sdf1*), a chemokine thought to be responsible for directional migration in the mouse embryo (Ara et al., 2003; Molyneaux et al., 2003) (Fig. S4D). It is possible that these spatially localised supporting cells enable the maturation of Gld-PGCLCs to post-migratory stages of development as they begin to express not only DAZL but also GCNA1 (or GCNA), a marker of post-migratory PGCs *in vivo* (Enders and May, 1994) (Fig. S4E).

### Transcriptomic Gld-PGCLC characterisation

Given the general signature of PGC identity observed in Gld-PGCLCs, including the surprisingly mature status of DAZL and GCNA1 expression, we wanted to compare our Gld-PGCLCs with known populations of PGCs, both *in vivo* and *in vitro*, at the transcriptomic level. To do this, we sorted Blimp1:mVenus^+^, SSEA1^+^, PECAM1^+^ and Stella:eCFP^+^ cells from 120 h gastruloids and performed 10x single-cell RNA-sequencing (scRNA-seq) (Materials and Methods; Fig. S5A). Once integrated into a single 120 h dataset, we identified eight distinct clusters of cell identities (clusters 0 to 7), including five that we denoted to be putative PGCLCs owing to expression of genes including *Dppa3*, *Dppa4*, *Dppa5a*, *Nanog*, *Oct4*, *Sox2*, *Blimp1* and *Tfap2c* (Fig. S5B). In addition, some cells within these clusters also expressed genes including *Dazl*, *Ddx4* and *Tex14*, which are known markers of later-stage PGCs in the mouse embryo. Although each sorted population contributed to these PGC-like clusters, we also noted additional populations, including a putative endoderm-like population (cluster 6), an endothelial population (cluster 7) and a mesodermal population, including somitic cell types (cluster 5), that were apparent in our data (Fig. S5B,C). To further confirm that our sorting strategies indeed captured the population of Gld-PGCLCs, we compared our data with extant mouse gastruloid scRNA-seq data (van den Brink et al., 2020) and confirmed a high degree of concordance between both PGCLC populations (Fig. S5D-E). We therefore filtered our cells using the previously defined PGCLC population from mouse gastruloid scRNA-seq data (van den Brink et al., 2020) for all downstream analysis.

**Fig. 5.**
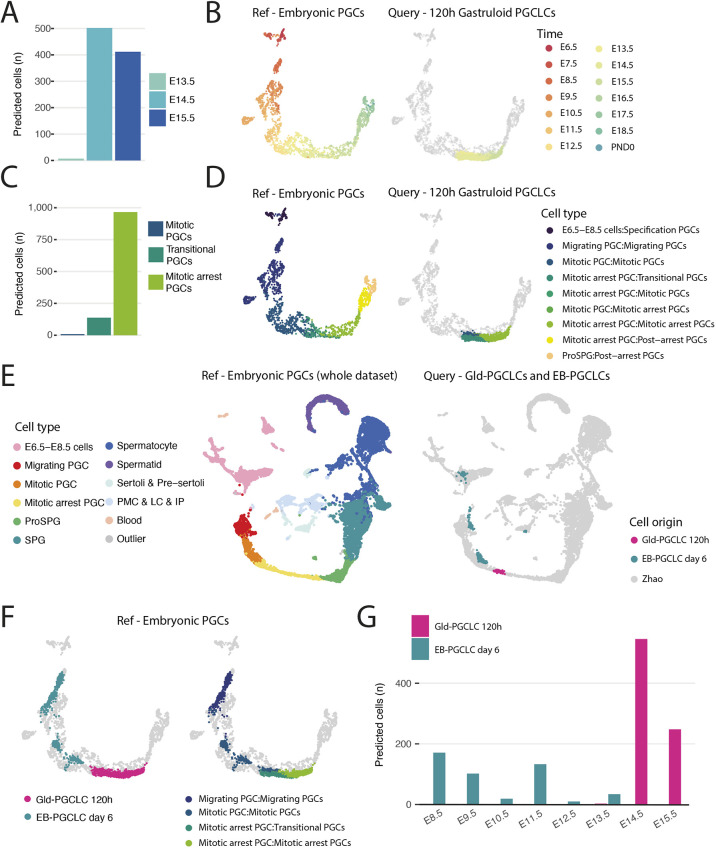
**Single-cell transcriptomic comparison between expression data from Gld-PGCLCs and an *in vivo* PGC dataset.** (A,C) Quantification of label transfer prediction from Gld-PGCLCs (0.6+ maximum prediction score) in terms of embryonic time (A) and cell stage (C). (B,D) UMAP of PGC-only cell types from Zhao et al. (2021), in terms of time (B) and stage (D) with Gld-PGCLCs embedded. (E) UMAP projection of Gld-PGCLCs (0.9+ maximum prediction score) and published EB-PGCLCs (Ramakrishna et al., 2022) into the *in vivo* UMAP of the full dataset. (F) Comparison of UMAP projection of Gld-PGCLCs and published EB-PGCLCs onto the *in vivo* PGC dataset, by origin (left) and cell state (right). (G) Frequency of cell transfer labels from EB-PGCLCs or Gld-PGCLCs (0.6+ maximum prediction score) onto the *in vivo* PGC dataset, by embryonic time point. IP, interstitial progenitors; LC, Leydig cells; PMC, peritubular myoid cells; PND0, postnatal day 0; ProSPG, pro-spermatogonia; SPG, spermatogonia.

One of our major questions was whether these cells were equivalent to *in vivo* PGC types, and if so, which developmental timepoint was best matched by the *in vitro* Gld-PGCLCs. To assess this, we projected our Gld-PGCLCs onto a well-characterised and extensive map of *in vivo* germ cell development between E6.5 and adulthood (8-10 weeks) at 28 sampled timepoints from Zhao and colleagues (2021). Surprisingly, we found a very close match between our Gld-PGCLC cells and *in vivo* PGCs at the mitotic and mitotic arrest PGC stages of development, which were found in E13.5- to E15.5-stage embryos (Fig. 5A-D). This is particularly remarkable given that traditional EB-derived PGCLCs are thought to stall at E9.5-E10.5 stages (Hayashi et al., 2011). We therefore directly compared our Gld-PGCLC dataset with a published single-cell dataset from EB-derived PGCLCs at day 6 (Ramakrishna et al., 2022) with the *in vivo* PGC dataset. We found that the EB-PGCLCs were relatively heterogenous and their projection spanned cell types from specification PGCs to migrating PGCs and as late as mitotic PGCs (E8.5 to E13.5), whereas our Gld-PGCLCs were more homogeneous and clearly more advanced on the projection, and particularly approximated mitotic-arrest PGCs (E13.5 to E15.5; Fig. 5E-G).

To further investigate this timeline of Gld-PGCLCs, we performed a small-scale time-course experiment of Gld-PGCLCs from gastruloids at 96, 120 and 144 h timepoints (Fig. S6A). These data showed a clear temporal progression from earlier to later PGC signatures and correspondingly mapped most strongly to migratory PGCs at 96 h, a mix of migratory, mitotic and mitotic-arrest PGCs at 120 h, and mitotic-arrest PGCs at 144 h (Fig. S6B), thereby confirming that the temporal differentiation of Gld-PGCLCs matches that of embryonic PGCs. Of note, a small population of sorted Gld-PGCLCs appeared to have label transfer of very late germ cell states (such as spermatid states), but a closer examination of marker gene expression in these populations identified likely contaminating mesodermal and endodermal cell populations (Fig. S6C), whereas the remaining cellular populations corresponded well to the PGCLC identity, and expressed many known markers of PGC states, additionally validating their identity as PGCLCs (Fig. S6D).

Likewise, projecting our Gld-PGCLCs onto the EB-PGCLC dataset from Ramakrishna et al. (2022) that includes embryonic PGCs from E10.5 and E13.5 embryos as well as naïve ESCs and primed epiblast-derived stem cells (EpiSCs), showed that our Gld-PGCLCs were mapped most frequently to embryonic E13.5 PGCs and sometimes to E10.5 PGCs, but not to the ESC or EpiSC populations, thereby ruling out that they are residual pluripotent cells, as has recently been proposed in mouse gastruloids (Suppinger et al., 2023). In addition, all sampled gastruloid timepoints matched well with our initial projections that identified that Gld-PGCLCs are equivalent to E14.5-E15.5 PGCs *in vivo* (Fig. S6F).

Altogether, this transcriptomic analysis of Gld-PGCLCs, alongside the observation of DAZL protein expression, epigenetic remodelling and cell morphological behaviours, suggests that gastruloids might enable the development of more mature PGC-like states *in vitro*, without the need for additional gonadal co-culture.

### Endogenous signalling control of Gld-PGCLC specification

As no exogeneous manipulation of the gastruloids was performed that might particularly bias towards a germ cell-like fate, we hypothesised that Gld-PGCLC specification and maturation must be coordinated by local, self-organised signalling feedback mechanisms between populations of cells present in the gastruloid. We therefore sought to manipulate the endogenous signalling environment of gastruloids and examine the resultant effect on the Gld-PGCLC population to better understand how these endogenous signals were acting.

**Fig. 6.**
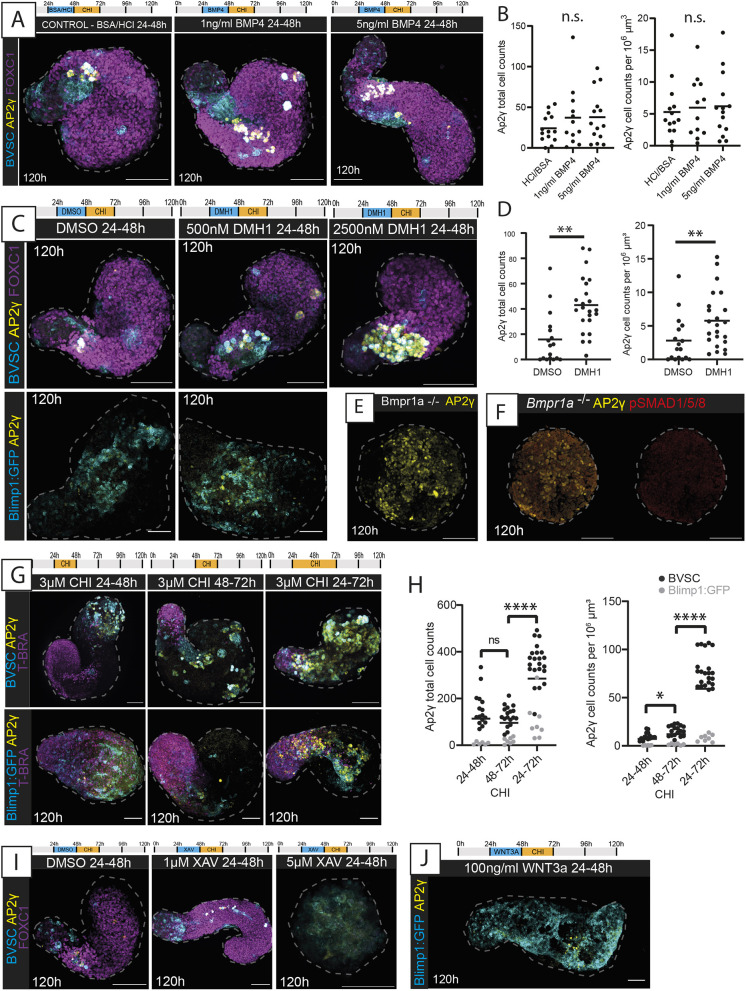
**BMP and Wnt signalling modulation in Gld-PGCLCs.** (A) Maximum-projection images of BVSC gastruloids following BMP4 application at the timepoints and concentrations indicated. In BVSC gastruloids, Blimp1:mVenus is membrane-targeted, whereas Stella:eCFP is found throughout the cell. (B) Quantification of AP2γ^+^ cells in the conditions indicated from BVSC and Blimp1:GFP gastruloids at 120 h. (C) Maximum-projection images of gastruloids following BMP inhibition by DMH1 application at the timepoints and concentrations indicated. (D) Quantification of AP2γ^+^ cells following DMSO or 500 nM DMH1 treatment, from BVSC and Blimp1:GFP gastruloids at 120 h. (E) Gastruloids made from the *Bmpr1a*^−/−^ cell line, showing aberrant morphology with lack of elongation and significant numbers of AP2γ^+^ cells. (F) Absence of pSMAD1/5/8 in *Bmpr1a*^−/−^ gastruloids at 120 h. (G) Maximum-projection images of gastruloids exposed to different timings of CHIR-99021 (CHI) application as indicated. (H) Quantification of AP2γ^+^ cells in the conditions indicated, from BVSC and Blimp1:GFP gastruloids at 120 h. (I) Maximum-projection images of BVSC gastruloids exposed to XAV939 (XAV) to inhibit Wnt signalling. (J) Maximum-projection image of a Blimp1:GFP gastruloid exposed to WNT3A at the timepoint shown. Black bars (B,D,H) represent the mean average. Dashed lines, morphological gastruloid outlines from Hoechst staining. Images are representative of 4-22 gastruloids per panel. Scale bars: 100 µm. n.s., no significant differences; *P<0.05; **P<0.01; ****P<0.0001 (unpaired, two-tailed *t*-test with Welch's correction).

We initially focussed on the BMP signalling pathway, as it has been reported to be required for PGC specification *in vivo* (Lawson et al., 1999; Ying et al., 2000, 2001; Chang and Matzuk, 2001; Ying and Zhao, 2001) and *in vitro* (Ohinata et al., 2009; Hayashi et al., 2002), although this has been brought into question by recent reports (Senft et al., 2019; Morgani and Hadjantonakis, 2021). Surprisingly, we found that addition of the BMP4 ligand did not lead to any significant increase in Gld-PGCLC numbers compared with those in control gastruloids (Fig. 6A,B; Fig. S7A,B). Likewise, no colocalisation of phosphorylated SMAD1/SMAD5/SMAD8 (a synonym of SMAD9) (pSMAD1/5/8) was found with AP2γ at 24 h in BVSC or 48 h in Blimp1:GFP gastruloids (Fig. S7C,D), and, in general, very little pSMAD1/5/8 was detected in the gastruloids until 96 h, at which timepoint the distribution was polarised towards the anterior pole but was never observed to colocalise with AP2γ (Fig. S7C,D). This is consistent with spatial transcriptomics data that reported an anterior bias of BMP signalling in gastruloids from late stages (Turner et al., 2017; Moris et al., 2020), but implies that downstream BMP signalling might not be active in the Gld-PGCLCs themselves. Indeed, addition of the small-molecule BMP inhibitor dorsomorphin homolog 1 (DMH1; a selective inhibitor of ALK2) to gastruloids from 24 to 48 h did not produce discernible morphological differences in axial elongation compared with that in the DMSO control, and both contained AP2γ/Stella-positive cells (Fig. 6C). However, a significant increase was found in absolute AP2γ^+^ cell count (*P*=0.0003; mean±s.d.=43±22.52 cells per gastruloid) and the proportion of AP2γ^+^ cells relative to gastruloid volume (*P*=0.0191, 5.78±4.2 cells per gastruloid) in gastruloids exposed to DMH1 (Fig. 6C,D). Consistent with this, higher concentrations of DMH1 resulted in further significant increases in AP2γ^+^ cell count (Fig. 6C; Fig. S8A-C), and LDN 193189 [LDN; an BMPR1A inhibitor] treatment likewise did not inhibit Gld-PGCLC formation (Fig. S8A).

To further explore this surprising relationship between BMP signalling and Gld-PGCLC specification, we generated gastruloids from *Bmpr1a*-null mESCs (Di-Gregorio et al., 2007). These gastruloids did not elongate (Fig. 6E,F), perhaps consistent with the reported reduced Nodal/activin signalling found in *Bmpr1a*-null embryos (Di-Gregorio et al., 2007) and the requirement for Nodal signalling in symmetry breaking and elongation in gastruloids (Turner et al., 2017). However, they did show evidence of differentiation towards endodermal, mesodermal and ectodermal populations (Fig. S8D-I). Surprisingly, they also contained AP2γ-expressing cells (Fig. 6E; Fig. S8D) in significantly higher proportions than observed in non-mutant Blimp1:GFP/BVSC gastruloids (Fig. S8D). Taken together, these results suggest that BMP signalling is not strictly required for PGCLC specification in the gastruloid model, and indeed may even have a repressive effect on the Gld-PGCLC fate, at least at the timepoints assessed here.

We then turned our attention to the Wnt signalling pathway, which has also been proposed to support PGC specification *in vivo* (Ohinata et al., 2009; Aramaki et al., 2013). As the standard gastruloid protocol includes a 24 h pulse of CHIR-99021 (herein, CHI; an inhibitor of GSK3β or GSK3B) between 48 and 72 h post-aggregation, we decided to modulate the time interval of addition of CHI to examine the effect on Gld-PGCLCs. Moving the CHI addition 24 h earlier altered gastruloid morphology but did not inhibit the presence of AP2γ^+^ cells (Fig. 6G). However, extending the CHI exposure to between 24 and 72 h post-aggregation resulted in significant (*P*<0.0001) increase in AP2γ-positive cells in both absolute (285.4±138.1) and relative (59.13±34.39) cell numbers, although we noted a line-specific difference between the Blimp1:GFP and BVSC lines (Fig. 6G,H). The increase in AP2γ^+^ cells was specific to this time window, as CHI treatment for an equivalently prolonged period of 48 h between 0 and 48 h post-aggregation in the BVSC gastruloids did not significantly alter the AP2γ^+^ cell number (*P*=0.43), even though it did result in clear morphological changes (Fig. S9A), and later addition of CHI (72-96 h) led to a significant decrease in Gld-PGCLCs (Fig. S9B,C). However, although changing the timing of CHI exposure had an obvious effect on Gld-PGCLC numbers, altering the concentration of CHI between 48 and 72 h did not change the numbers of AP2γ^+^ cells relative to the total gastruloid (Fig. S9D-G). Taken together, these results suggest that gastruloid PGCLCs are sensitive to Wnt signalling modulation, but also that this sensitivity occurs within a specific temporal window, in a time-dependent but not concentration-dependent manner.

In addition, it is likely that endogenous as well as exogeneous Wnt signalling may drive PGCLC formation in gastruloids. Gastruloids without a CHI pulse still contained AP2γ-expressing cells (Fig. S9H,I), but BVSC gastruloids exposed to Wnt inhibition by addition of XAV393 (XAV) resulted in loss of Gld-PGCLCs (*P*=0.033, Fig. 6I; Fig. S9I). Additionally, the supplementation of 500 ng/ml WNT3A led to a significant (*P*=0.0023) increase in Gld-PGCLCs in the BVSC gastruloids (Fig. S9H,I), although addition of 100 ng/ml WNT3A on Blimp1:GFP gastruloids did not lead to significant changes (Fig. 6J). Taken together, these observations suggest that Wnt signalling is indeed necessary for the specification of Gld-PGCLCs and that gastruloids are particularly sensitive to the effect of this pathway between 24 and 72 h post-aggregation.

**Fig. 7.**
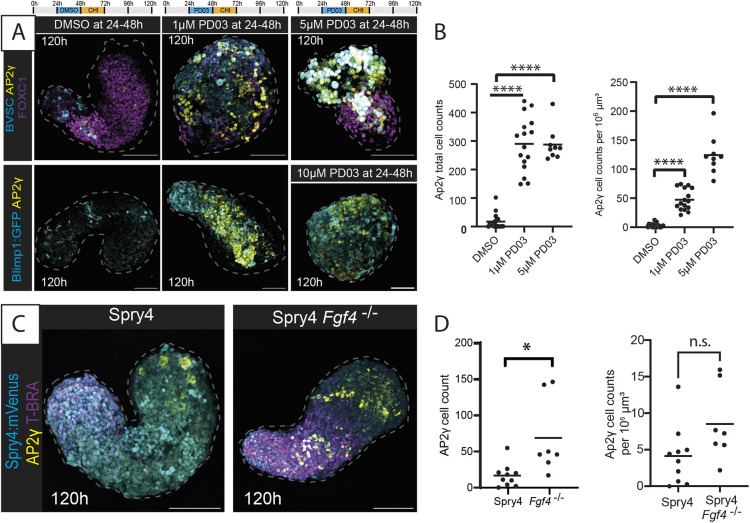
**FGF signalling modulation in Gld-PGCLCs.** (A) Maximum-projection images of gastruloids exposed to PD0325901 (PD03) to inhibit FGF signalling. In BVSC gastruloids, Blimp1:mVenus is membrane-targeted, whereas Stella:eCFP is found throughout the cell. (B) Quantification of AP2γ^+^ cells in the conditions indicated, from BVSC and Blimp1:GFP gastruloids at 120 h. (C) Maximum-projection images of Spry4:mVenus *Fgf4*^−/−^ gastruloids at 120 h. (D) Quantification of AP2γ^+^ cells in non-mutant Spry4:Venus gastruloids and in Spry4:mVenus *Fgf4*^−/−^ gastruloids at 120 h. Dashed lines, morphological gastruloid outlines from Hoechst staining. Images are representative of 3-15 gastruloids per experiment. Scale bars: 100 µm. n.s., no significant differences; *P<0.05; ****P<0.0001 (unpaired, two-tailed t-test with Welch's correction).

Finally, we turned our attention to the FGF signalling pathway, as an *in vitro* study found that FGF inhibition during mesodermal induction resulted in the formation of mouse PGCLCs (Kimura et al., 2014). Phosphorylated ERK1/ERK2 (pERK) was observed sporadically with no discernible spatial polarisation in 24 or 48 h gastruloids and neither was it specifically associated with AP2γ-positive cells (Fig. S10A). Perturbation of the FGF pathway by addition of the FGF signalling inhibitor PD0325901 (PD03), between 24 and 48 h resulted in a marked increase in AP2γ-expressing cells, accompanied by loss of gastruloid elongation and disruption of FOXC1, a marker of the anterior mesoderm (Fig. 7A). The total AP2γ-expressing cell count for PD03-treated gastruloids (290.3±95.45 cells) was significantly (*P*<0.0001) higher than that of the DMSO control and increased in a concentration-dependent manner when adjusted for gastruloid volume (Fig. 7A,B; Fig. S10B,C). This observation was independent of CHI, as an increase in AP2γ expression was also observed when PD03 was added to gastruloids in the absence of the CHI pulse (Fig. S10B,C). The FGF inhibition-induced increase in AP2γ was also timeframe specific, with the largest change in AP2γ number following PD03 addition between 24 and 48 h (Fig. S10D).

To further explore the role of FGF signalling on Gld-PGCLC specification, we made gastruloids from cells containing a fluorescent reporter of Spry4 (Spry4:Venus), the downstream target of the FGF pathway, as well as this same reporter line with FGF4 knockout (Spry4:Venus; *Fgf4*^−/−^) (Morgani et al., 2018). Spry4:Venus expression was found to be biased towards the more posterior end of the gastruloids, consistent with a posterior FGF signalling gradient in gastruloids (Turner et al., 2017) and in the gastrulation-stage embryo (Yamaguchi et al., 1994; Oki et al., 2010), and, similar to our pERK staining patterns, Spry4 reporter expression did not overlap specifically with the AP2γ^+^ population (Fig. 7C). However, gastruloids generated from the *Fgf4* mutant cells had a significant increase in the number of AP2γ-positive cells (*P*=0.0398; Fig. 7D), akin to our results with small-molecule inhibition of this pathway. Although we cannot rule out that these FGF-modulated AP2γ-positive cells show differences to Gld-PGCLCs in the absence of exogenous FGF signalling, these results are suggestive of potential Gld-PGCLC sensitivity to FGF signalling levels, which should be investigated in future studies. Together, these signalling modulation experiments suggest that there are specific time windows that are sensitive to signalling pathway perturbation in mouse gastruloids, which might correspond to times at which cellular populations undergo cell-fate decisions or emerge as new cell types, and particularly implicate the Wnt and FGF pathways as key modulators of Gld-PGCLCs in gastruloids.

## DISCUSSION

We have shown that gastruloids generated from established PGC reporter lines contain a population of cells, which we call Gld-PGCLCs, that display key features of PGCs, including co-expression of pluripotency and PGC-associated markers. Our findings, combined with those by others (van den Brink et al., 2020; Veenvliet et al., 2020; Vianello and Lutolf, 2020 preprint; Cermola et al., 2021; Hashmi et al., 2022), demonstrate that Gld-PGCLCs appear to be a general feature present in mouse gastruloids, despite the fact that gastruloids self-organise in the absence of extra-embryonic tissues (Turner et al., 2017). Our results have shown that gastruloids are able to specify a population of PGCLCs and support the continued maturation of this population towards late-PGC identities, dynamically recapitulating many aspects of their *in vivo* counterparts in gene/protein expression, epigenetic changes and cell behaviour.

In addition, the Gld-PGCLCs generated here showed advanced maturation equivalent to ∼E14.5-stage *in vivo* PGC development, which far surpasses traditional EB-PGCLC approaches that are believed to stall at approximately E9.5-E10.5 stages (Hayashi et al., 2011). One example of this is in the expression of DAZL, a late germ cell marker that is required for germ cell determination (Gill et al., 2011; Nicholls et al., 2019), which Gld-PGCLCs express at 120 h but is not typically reached in EB-PGCLCs (Hayashi et al., 2011), except in the presence of additional expansion factors such as forskolin and rolipram (Ohta et al., 2017). It is likely that the close association of Gld-PGCLCs with neighbouring tissues in gastruloids, including the early primitive streak-like domain, the epithelial endodermal tract and the GATA4^+^ anterior niche cells, strongly supports the notion that mouse gastruloids benefit from organised co-development of Gld-PGCLCs alongside somatic populations. This could potentially explain their apparent maturation, as local endogenous signalling alongside dynamic cell movements might optimise the developmental time course of these cells towards developmentally faithful fates (Cooke and Moris, 2021). However, it is indeed surprising that Gld-PGCLCs are able to reach states equivalent to embryonic E14.5 PGCs by 144 h, given that previous studies have suggested that gastruloids at this experimental timepoint are overall most similar to ∼E9.5 stages (Beccari et al., 2018). It is possible that this observation therefore reveals potential intrinsic properties of PGC(LC)s: in the embryo, their development might take additional time, for instance, incorporating the need to traverse long distances to reach the incipient gonads, and yet the PGC(LC)s may already be competent to reach mitotic-arrest stages given the right environment in a simplified *in vitro* system. However, further studies would be required to test this hypothesis.

Our perturbation experiments likewise challenge the role of different signalling pathways in mouse PGC specification. Although BMP signalling has been proposed to principally mediate initial specification of the PGC lineage (Ohinata et al., 2009; Lawson et al., 1999), we found little evidence that BMP is required for Gld-PGCLC specification. These results are directly comparable with those performed by Morgani and Hadjantonakis (2021), who similarly showed that PGCLCs can be induced in *Bmpr1a*^−/−^ EBs, and indeed that the proportion of AP2γ^+^ PGCLCs increases in this case. Taken together, such results challenge the notion that BMP signalling is directly required for PGC(LC) induction. Instead, Wnt and FGF signalling appear to play a greater role in determining the germline-to-soma balance of cell-type proportions in gastruloids. It is possible that BMP signalling is a required feature of mouse embryonic PGC specification primarily because of its role in the extra-embryonic-to-embryonic signalling cascades that are necessary to localise the site of presumptive PGC specification to the proximal posterior epiblast (Lawson and Hage, 1994; Tam and Zhou, 1996; Yoshimizu et al., 2001; de Sousa Lopes et al., 2007). In gastruloids lacking extra-embryonic tissues, the competence of the cells to form PGCLCs is likely to be global rather than localised, similar to experiments isolating the epiblast from the visceral endoderm and extra-embryonic ectodermal tissues (Ohinata et al., 2009; Yoshimizu et al., 2001). However, unlike those early epiblast isolation assays, in this case, the time window of competence appears to have shifted beyond the BMP-receptive stage to a Wnt-receptive stage, particularly between 24 and 72 h of the gastruloid protocol, consistent with similar timepoints in the mouse embryo, at about E5.75 to E6.75 (Ohinata et al., 2009; Aramaki et al., 2013; Bialecka et al., 2012). After this, FGF may well act to ‘fine-tune’ the number and balance proportions of PGCLCs, as has been shown across early cell-fate decisions (Krawchuk et al., 2013), and similar to its function in separating PGCs from the soma in the axolotl (Chatfield et al., 2014). Whether this observation is partly specific to the *in vitro* gastruloid context or reflects a more general feature of mouse PGC specification and regulatory control remains to be seen.

Future research may help to unravel further the signalling mechanisms at play within such systems, including the cross-talk between signalling pathways, and the relationship between tissue types and signalling dependencies, potentially leading to answers to longstanding questions that still exist, such as how PGCs form in the proximal posterior epiblast along with multiple other cell types exposed to the same signalling environment, and what exactly determines the cell proportions (Cooke and Moris, 2021). In addition, gastruloids have more recently been generated from human pluripotent stem cells (Moris et al., 2020), so it would be very interesting to see whether these findings translate into human gastruloids, particularly given the current debate about the epiblast or amniotic origin of PGCs in the human embryo (Kobayashi and Surani, 2018).

Overall, our observations highlight the experimental tractability of *in vitro* embryo-like models to generate rare cell types within a native embryo-like context that opens a new route towards exploring exactly how tissue and cell interactions might mediate cell-fate specification in embryogenesis. In addition, the Gld-PGCLCs generated here represent an advanced maturation state that has not previously been achieved *in vitro* without the exogeneous application of PGC-specific maturation factors or gonadal co-culture. Both these features – their maturity and their inherent co-development – represent a unique advantage of using embryo-like model systems over traditional directed differentiation or disorganised EB systems, as cell types are specified in a manner that harnesses the mechanisms that are used by the embryo itself.

## MATERIALS AND METHODS

### Cell culture and maintenance

The following mESC lines were used: Blimp1:GFP (Ohinata et al., 2005) (kindly provided by Azim Surani, University of Cambridge, UK), Blimp1:mVenus Stella:eCFP (BVSC) (Ohinata et al., 2008) (kindly provided by Mitinori Saitou, ASHBi Institute for the Advanced Study of Human Biology, Kyoto, Japan), *Sox17*^−/−^ (as described below), *Foxa2*^−/−^ (Cernilogar et al., 2019) (kindly provided by Heiko Likert, Helmholtz Munich, Germany), *Bmpr1a*^−/−^ (Di-Gregorio et al., 2007) (kindly provided by Tristan Rodriguez, Imperial College London, UK), and Spry4:Venus and Spry4:Venus *Fgf4*^−/−^ (Morgani et al., 2018) (kindly provided by Christian Schroeter, Max-Planck-Institut für Molekulare Physiologie, Dortmund, Germany). All mESC lines were cultured in 2iLIF in N2B27 medium [NDiff227, Takara Bio, Y40002; supplemented with 3 µM CHIR-99021 (CHI), 1 µM PD0325901 (PD03) and 11 ng/ml mLIF (Merck Millipore ESG1106 and made in-house by the Department of Biochemistry, Cambridge University, UK)] on gelatinised (0.1% gelatin) tissue culture flasks or six-well plates kept in humidified incubators at 37°C and 5% CO_2_. Cells were passaged into new flasks or plates every two days with the medium exchanged daily.

### Generation of *Sox17*^−/−^ cell line

Cells were grown for at least two passages prior to transfection. Cas9/guide RNA (gRNA) targeting was used to generate strand breaks alongside homologous recombination with a targeting vector (Kim et al., 2007). An eGFP sequence was knocked in to both alleles of the *Sox17* gene by plasmid transfection. gRNAs were designed to target protospacer adjacent motif (PAM) sequences at the start and end of the protein coding sequence (Table S4). gRNAs were ligated into the PX459-Cas9 plasmid (Addgene, #62988) (Ran et al., 2013) after cleavage with BbsI (New England BioLabs, R0539S). The correct integration of the gRNAs was confirmed after cloning by Sanger sequencing using the hU6-F oligonucleotide (see Table S4). Cells were transfected with three plasmids [*Sox17-GFP* (Addgene #23334), *PX459-gRNA1* (made in-house by ligation of gRNA1 into PX459; see Table S4) and *PX459-gRNA2* (made in-house by ligation of gRNA2 into PX459; see Table S4)] by incubation with FuGene HD (Promega, E2311) following a previously described protocol (Mulas et al., 2019). Transfected cells were grown under selection with puromycin (Thermo Fisher Scientific, A1113803) and clones were picked for expansion. Genomic DNA was prepared from the primary clones for genotyping by PCR with the primers described in Table S4.

### Gastruloid generation

Gastruloids were prepared following a previously reported protocol (https://doi.org/10.1038/protex.2018.094). Briefly, mESCs were trypsinised and pelleted, the cell pellet was washed in PBS, the process was repeated, and the cells were resuspended in N2B27. The cells were counted and diluted to provide 300 cells per well, before pipetting into U-bottom suspension 96-well plates (Greiner), except in the case of BVSC cells, which were pipetted into cell-repellent, ultra-low-attachment 96-well plates (Greiner). Aggregates were incubated at 37°C and 5% CO_2_ in a humidified incubator. After 48 h, N2B27 medium supplemented with 3 µM CHI was added, and every subsequent 24 h, the medium was aspirated and replaced with fresh N2B27. Signalling modulation in gastruloids was performed through addition of small-molecule ligands or activators/inhibitors as indicated in the text and figure legends (Table S5).

### Immunostaining

Immunostaining was performed based on a previously published protocol (Baillie-Johnson et al., 2015). Gastruloids were collected and washed twice in PBS before fixing in 4% paraformaldehyde in PBS at 4°C (2 h to overnight on an orbital shaker). Gastruloids were washed three times with PBS to remove the paraformaldehyde and another three times with blocking buffer PBSFT [10% FBS (Biosera, FB-1090/500) and 0.2% Triton X-100 in PBS], before blocking in PBSFT for 1-2 h at 4°C on an orbital shaker. Primary antibodies (see Table S6) were added in PBSFT and incubated overnight at 4°C on an orbital shaker. A total of ten washes with PBSFT were performed, before secondary antibodies (diluted 1:500) (see Table S7) and Hoechst 33342 trihydrochloride trihydrate (Invitrogen Molecular Probes, H3570, 10 mg/ml solution in water, 16.2 mM) at 1:800 dilution were added and incubated overnight at 4°C on an orbital shaker. Three PBSFT washes followed by five PBT (0.2% FBS and 0.2% Triton X-100 in PBS) washes were performed. The gastruloids were then transferred to ScaleS4 tissue clearing solution [40% D-(−)-sorbitol, 10% glycerol, 4 M urea, 0.2% Triton X-100 and 20% DMSO] in a glass-bottomed dish, and incubated overnight at 4°C on an orbital shaker or mounted on coverslips before imaging.

For anti-5hmC immunostaining, the gastruloids were treated with 1 N HCl for 1 h at room temperature to expose the DNA prior to primary antibody addition. For the anti-phosphorylation antibodies, PBS in the solution buffers was replaced with TBS.

### Imaging

Confocal imaging was performed with either a Zeiss LSM770 or LSM880 inverted confocal microscope using a Plan-Apochromat 20×/0.8 DICII air objective, imaging 6 µm *z*-sections. Data were captured using the Zen software (Carl Zeiss Microscopy) and images were processed using ImageJ (FIJI) (Schindelin et al., 2012) to generate *z*-slice section images or *z*-stack maximum projections. The Hoechst channel, when not shown, was used to trace gastruloid outlines to show morphology.

Live imaging was carried out in environmental control units (humidified, 5% CO_2_, 37°C) using either widefield Nikon Inverted Eclipse Ti2 microscopes (15× or 20× ELWD objectives, GFP/YFP/mCherry triple filter) operated by open-source Micro manager software (Vale laboratory, University of California San Francisco, USA) or a Zeiss LSM880 NLO inverted multi-photon microscope (20× objective) operated by Zen software. The Chameleon laser in the multi-photon microscope was tuned to 880 nm, with filters 515/30 and 450/80 to detect GFP and CFP, and CFP only, respectively. Images were captured in single plane every 20 min for over 14 h on the Nikon microscope and *z*-stacks taken every 30 min for over 18 h on the multi-photon microscope.

### Image analysis

Expression profiles were generated in ImageJ by drawing a segmented line (120-pixel width for whole-gastruloid profiles or 20-pixel width for DAZL/NANOG-positive cells) from the posterior to anterior of *z*-stack maximum projections of the gastruloids (also used to determine length of gastruloids with the ‘Measure’ function), plotting the fluorescence profile using the ‘Plot Profile’ function, then normalising both the length and signal (against the Hoechst signal), before plotting in Prism (GraphPad) software.

Cell counting (parameter option: cell size=8) and gastruloid volume calculations were performed using the IMARIS software (Oxford Instruments), with gastruloid volumes calculated by creating a surface (surface smoothing 1.5, threshold 800-2000) on the Hoechst channel. Cell tracking and co-expression analysis was also performed using IMARIS software. Gastruloid tissue features and PGCLC clusters were assessed by eye in ImageJ. Means, standard deviations and significance (unpaired, two-tailed *t*-test with Welch's correction) were calculated in Prism.

### Doubling-time calculations

The doubling time of the PGCLCs was calculated based upon the mean cell numbers at each time point, using the following equations to first calculate the growth rate, then the doubling time between time points:

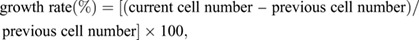


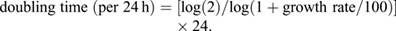


### Flow cytometry and cell sorting

Gastruloids were collected and washed twice in PBS before incubating at room temperature for 8 min in trypsin-EDTA (Thermo Fisher Scientific, 25300054) before quenching with 10% FBS in PBS. Cells were pelleted at 230 ***g*** for 5 min before being resuspended in filtered 1% FBS in PBS. The cell solution was passed through tube filter (35 µm; Corning, 352235) then counted and divided into tubes before antibody addition. Samples were incubated at 4°C on a rotator for 1 h, then centrifuged at 60 ***g*** for 5 min at 4°C. The supernatants were aspirated, the samples washed twice with filtered 1% FBS in PBS and transferred to chilled flow tubes. Cells were applied to a FACSAria Fusion III flow cytometer (BD Biosciences); flow cytometry was performed by the Francis Crick Flow Cytometry Science and Technology Platform (STP) staff. Data analysis was performed using FlowJo (BD Biosciences) software.

Sorting was based on the reporters Stella:eCFP, Blimp1:mVenus or PECAM1-PE and SSEA1-Alexa Fluor 647 (BD Biosciences, 561073, and BioLegend, 125607, respectively) antibodies. Sorted cells were transferred to DNA low-bind tubes and centrifuged at 300 ***g*** for 5 min at 4°C. The supernatant was aspirated and, using cut tips, 1 ml of chilled PBS pipetted up and down ten times. This was repeated twice more. After the final centrifugation step, the cells were resuspended in 200 µl chilled PBS and 800 µl of chilled 100% methanol was added dropwise with stirring. Fixed cells were stored at −80°C until ready for preparation for 10x scRNA-seq.

### 10x scRNA-seq

The sorted, fixed and frozen cells were thawed on ice for 5 min before centrifugation at 1000 ***g*** for 5 min at 4°C. The supernatant was carefully aspirated without disturbing the pellet, before resuspending the pellet in the appropriate volume of wash-resuspension buffer (3× SSC Buffer, Invitrogen, 15557-044) supplemented with 0.04% bovine serum albumin (Invitrogen, AM2616), 1 mM DL-dithiothreitol (DTT) solution (Sigma-Aldrich, 646563) and 0.2 U/µl Protector RNase inhibitor (Roche, 3335399001), to give 1000 cells/µl in 50 µl, or a minimum volume of 50 µl if it was not possible to obtain that concentration.

Quality control on the cells and counts were performed on a Luna FX7 cell counter (Logos Biosystems), prior to applying to 10x Chromium library preparation (10x Genomics), performed according to the manufacturer's instructions by the Advanced Sequencing Facility staff at the Francis Crick Institute. Single-cell libraries of 100 bp paired-end reads were pooled and sequenced using an Illumina NovaSeq 6000, carried out by the Advanced Sequencing Facility at the Francis Crick Institute.

### scRNA-seq analysis

FastQ files were quantified into expression matrices using Cell Ranger (v6.1.2; Zheng et al., 2017) using the 10x-provided refdata-gex-mm10-2020-A index. Seurat (v4.0.3; Hao et al., 2021) objects were created using the filtered matrix for each sorted population in R 4.1.1. Each population was filtered according to the number of reads, features and proportion of mitochondrial expression to remove low-quality cells. Quality-controlled datasets were integrated into timepoint datasets with Seurat (Stuart et al., 2019) and further into a time-course dataset. Datasets were scaled, projected and clustered using the first ten principal components for each sorted population or 15 principal components for the 120 h integrated dataset. For visualisation purposes, marker gene expression was shown by kernel density estimation using Nebulosa (Alquicira-Hernandez and Powell, 2021).

Published datasets were reprocessed using Seurat v4.0.3 from either the counts matrix of a Seurat object or output of Cell Ranger. To isolate PGCs from the dataset from Zhao et al. (2021), only cells for which the author-determined cell type included ‘PGC’ were included. Where possible, the same cell barcodes, variable features and dimensionality were used when reprocessing the published datasets and any published cell metadata were included. Qualitative comparison between the published and recalculated uniform manifold approximation and projections (UMAPs) reassured us that the structure in the reference data was preserved in our reprocessed objects. For visualisation, UMAP coordinates were reflected to preserve left-to-right time progression, where possible.

Reference and query datasets were subsequently analysed using Seurat (Hao et al., 2021) to transfer labels of published data onto the query data and embed the query data into the reference UMAP. We first used the van den Brink dataset (van den Brink et al., 2020) as a query and transferred the cell type label onto the 120 h dataset, which was subsequently filtered for cells that were most-likely PGC-like. The dataset from Zhao et al. (2021) was used as a reference for the 120 h PGCLCs and PGC cells identified in Ramakrishna et al. (2022) as ‘cluster 5 excluding E10.5’. From this comparison, both cell type labels (‘cell type 1’ and ‘cell type 2’) as well as time points were transferred. The same methods were used to project the complete time-course dataset without additional filtering for predicted cell types.

## Supplementary Material

10.1242/develop.201790_sup1 Supplementary information
