# Supplementary material for: Gastruloid-derived primordial germ cell-like cells develop dynamically within integrated tissues
Source: Development. 2023 Sep 11;150(17):dev201790. doi: 10.1242/dev.201790 (PMC10508693; doi:10.1242/dev.201790)
Supplement: Supplementary information [file develop-150-201790-s1.pdf]

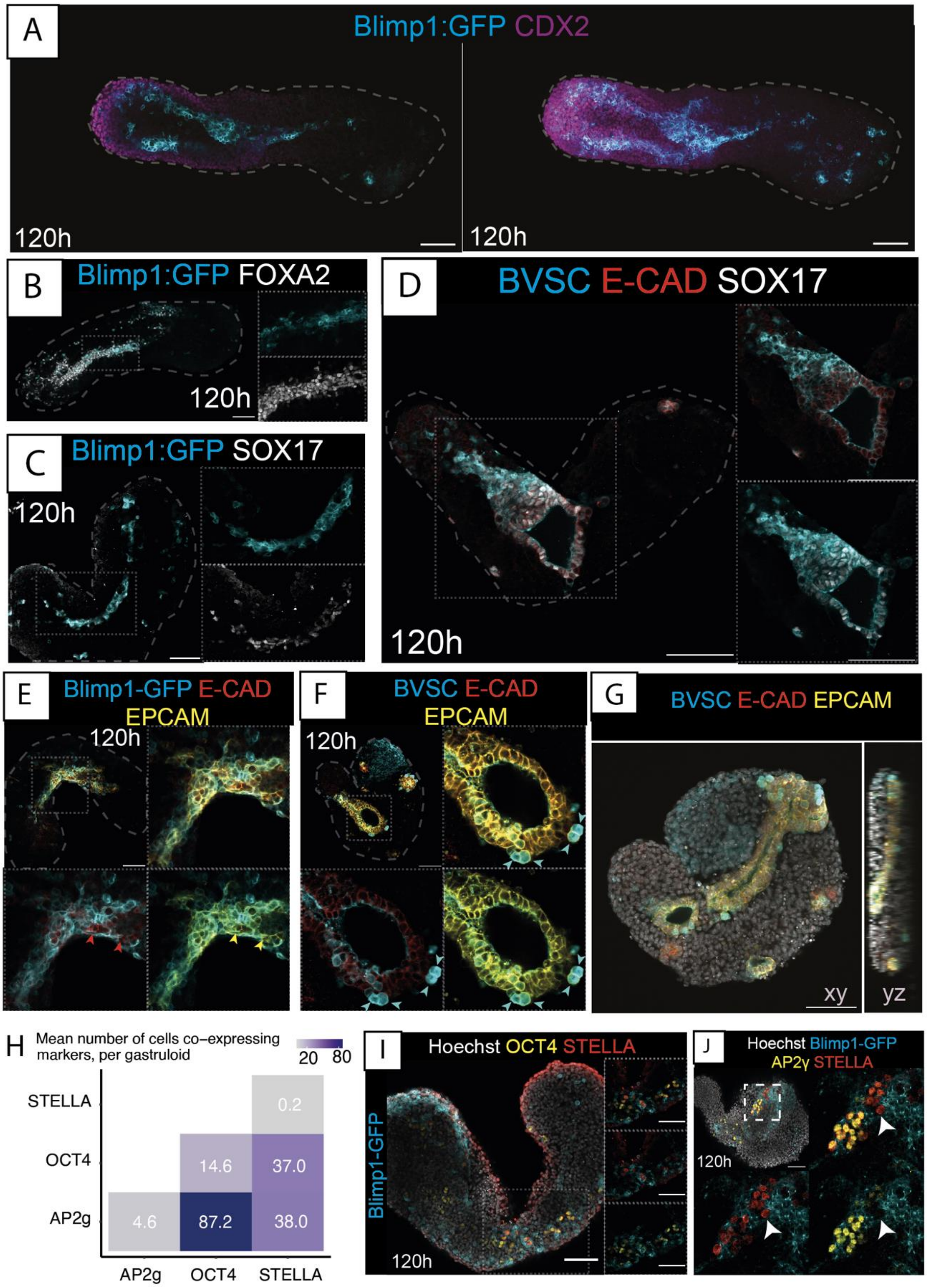

Supplementary Figure 1

Development • Supplementary information

Fig. S1. Characterisation of Gld-PGCLCs and endodermal tracts. (A) Z-slice (left) and maximum projection (right) of Blimp1-GFP gastruloid at 120h. **(B-D)** Expression of endodermal markers, FOXA2 **(B)** and SOX17 in Blimp1-GFP **(C)** and BVSC **(D)** gastruloids. **(E-G)** Expression of EpCAM in Blimp1-GFP **(E)** and BVSC **(F)** gastruloids. Cyan arrowheads, STELLA+ cells. **(G)** Z slice showing internal localisation of the EPCAM+, E-CADHERIN (E-Cad)+ tubular structure (left) and z-projection (right). **(H)** Quantification of number of cells co-expressing each pair of proteins, as indicated by colour scale. **(I)** Heterogeneous co-staining of STELLA with OCT4 and AP2γ in Blimp1-GFP gastruloids. White arrowhead, example of a STELLA+ AP2γ- cell. Insets, higher magnification images; Dashed line, morphological gastruloid outline from Hoechst staining; Dotted line, magnification region. Scale bars, 100 µm.

Development • Supplementary information

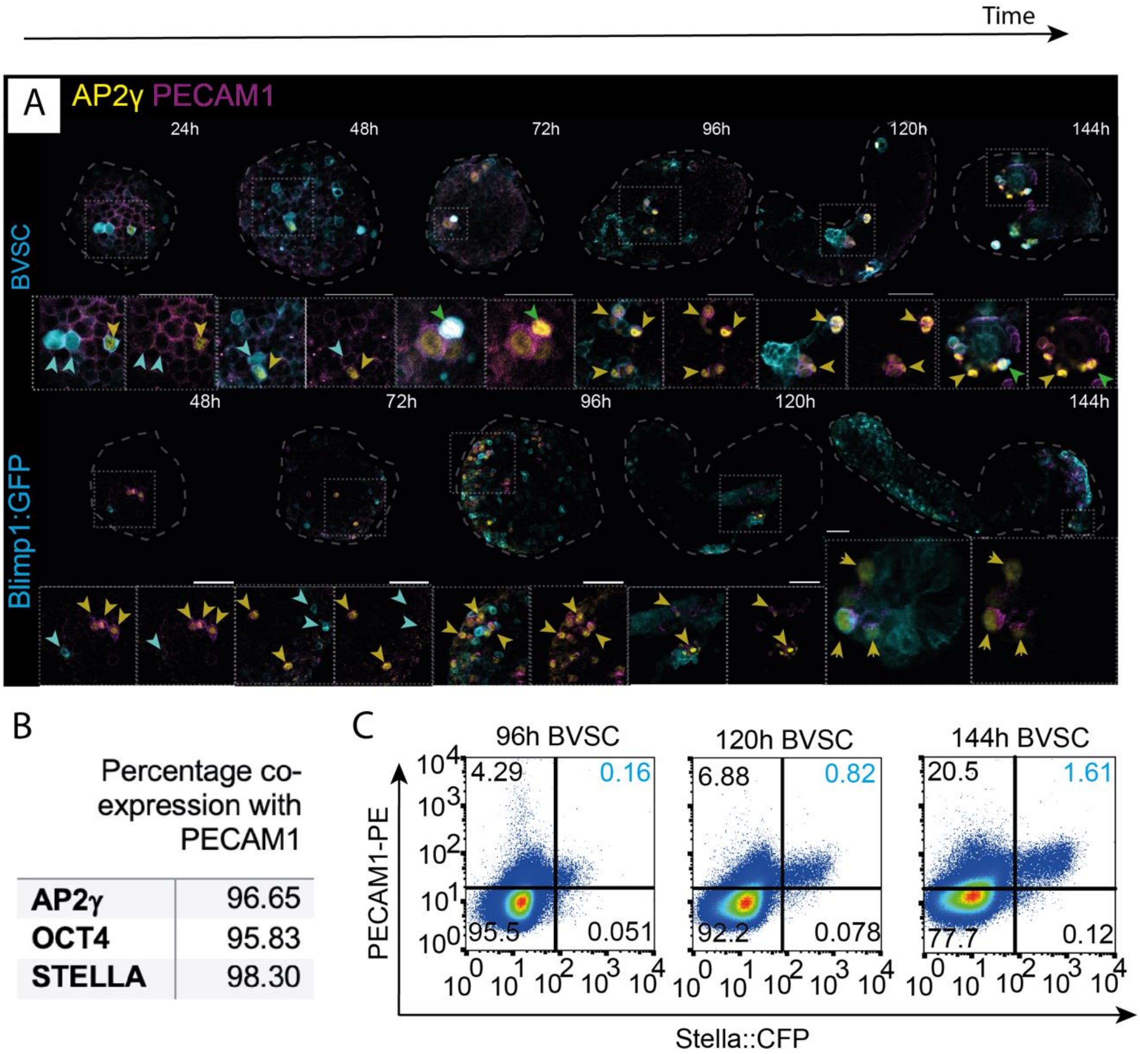

Supplementary Figure 2

Fig. S2. Time course analysis of Gld-PGCLCs as indicated by PECAM1 staining. (A) Time course of BVSC and Blimp1-GFP gastruloids from 24-144h, showing co-expression of PGCLC markers. Cyan arrowheads, STELLA+ cells; Yellow arrowheads, AP2γ+ cells; Green arrowheads, STELLA+ AP2γ+ cells. Insets, higher magnification images; Dashed line, morphological gastruloid outline from Hoechst staining; Dotted line, magnification region. Scale bars, 100 µm. **(B)** Quantification of BVSC cells expressing protein indicated that also express PECAM1, as percentages, at 120h. **(C)** Flow cytometry analysis of Stella:eCFP from BVSC line and PECAM1, where dot-plot colour-bar shows data density. Numbers in graph show percentage occupancy of each quadrant.

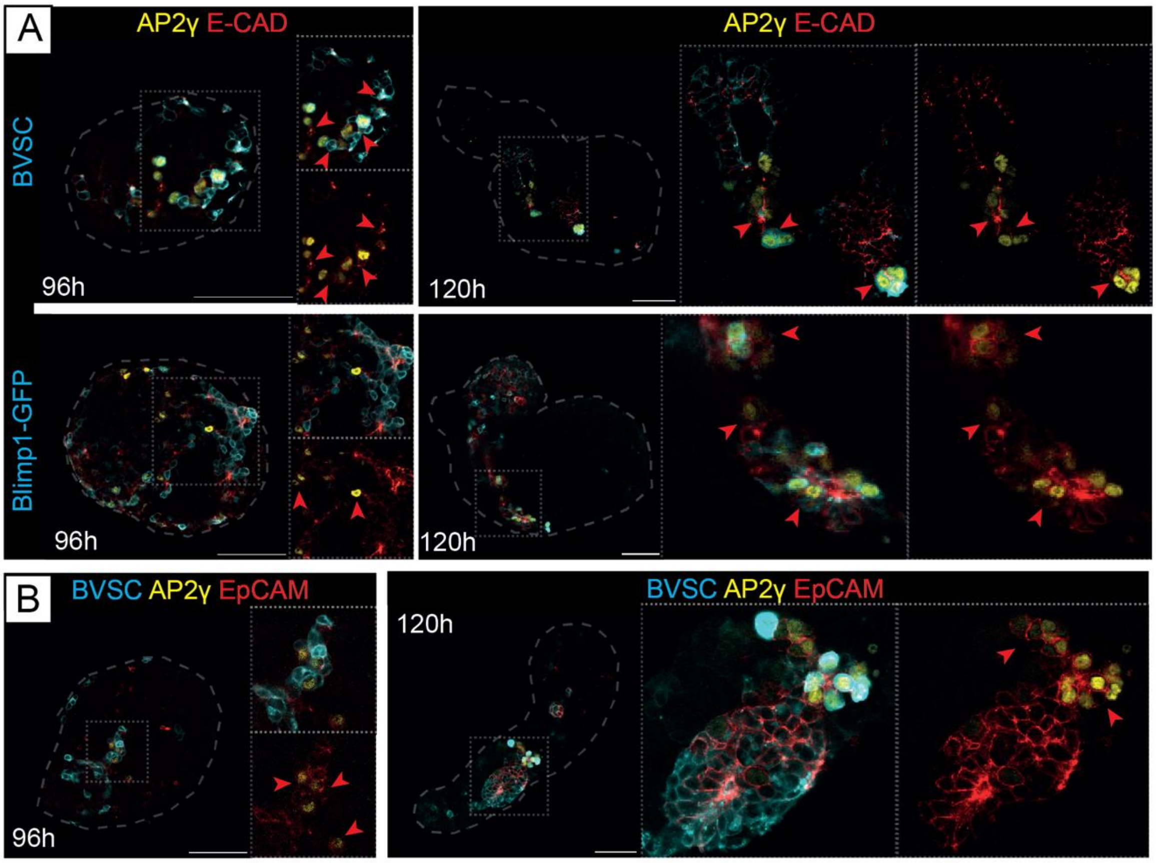

Supplementary Figure 3

Fig. S3. Expression of cell membrane markers on Gld-PGCLCs and endodermal tract cells. (A) Expression of E-Cadherin (E-Cad) in BVSC and Blimp1-GFP gastruloids. Red arrowheads, E-CAD on Gld-PGCLCs and neighbouring cells. **(B)** Expression of EpCAM in BVSC gastruloids. Red arrowheads, expression of EpCAM on Gld-PGCLCs and neighbouring cells. Insets, higher magnification images; Dashed line, morphological gastruloid outline from Hoechst staining; Dotted line, magnification region. Scale bars, 100 µm.

Development • Supplementary information

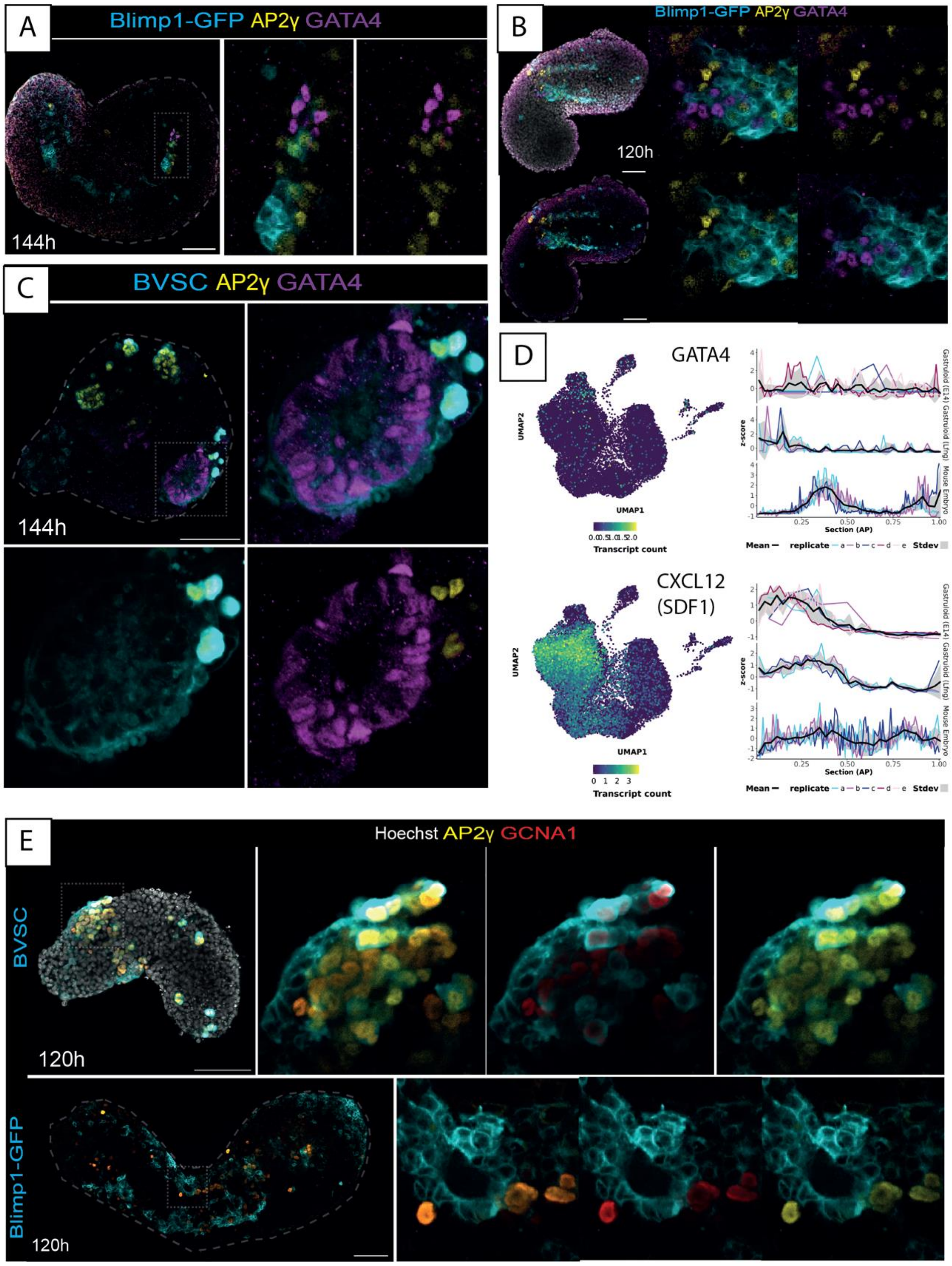

Supplementary Figure 4

Fig. S4. Anterior Gld-PGCLC cluster associated gene expression. (A-C) Expression of GATA4 in anterior regions adjacent to Gld-PGCLCs, in Blimp1-GFP **(A, B)** and BVSC **(C)** gastruloids. **(D)** A published gastruloid dataset shows expression of *Gata4* and *Cxcl12* genes (left),

Development • Supplementary information

particularly localised to anterior end as evidenced by tomo-sequencing (right). Individual lines represent different sample replicates, black line represents the average profile, and grey ribbon represents the standard deviation (Stdev). See [1] for further details. **(E)** Expression of GCNA1 particularly in AP2γ+ Gld-PGCLCs. Insets, higher magnification images; Dashed line, morphological gastruloid outline from Hoechst staining; Dotted line, magnification region. Scale bars, 100 µm.

Development • Supplementary information

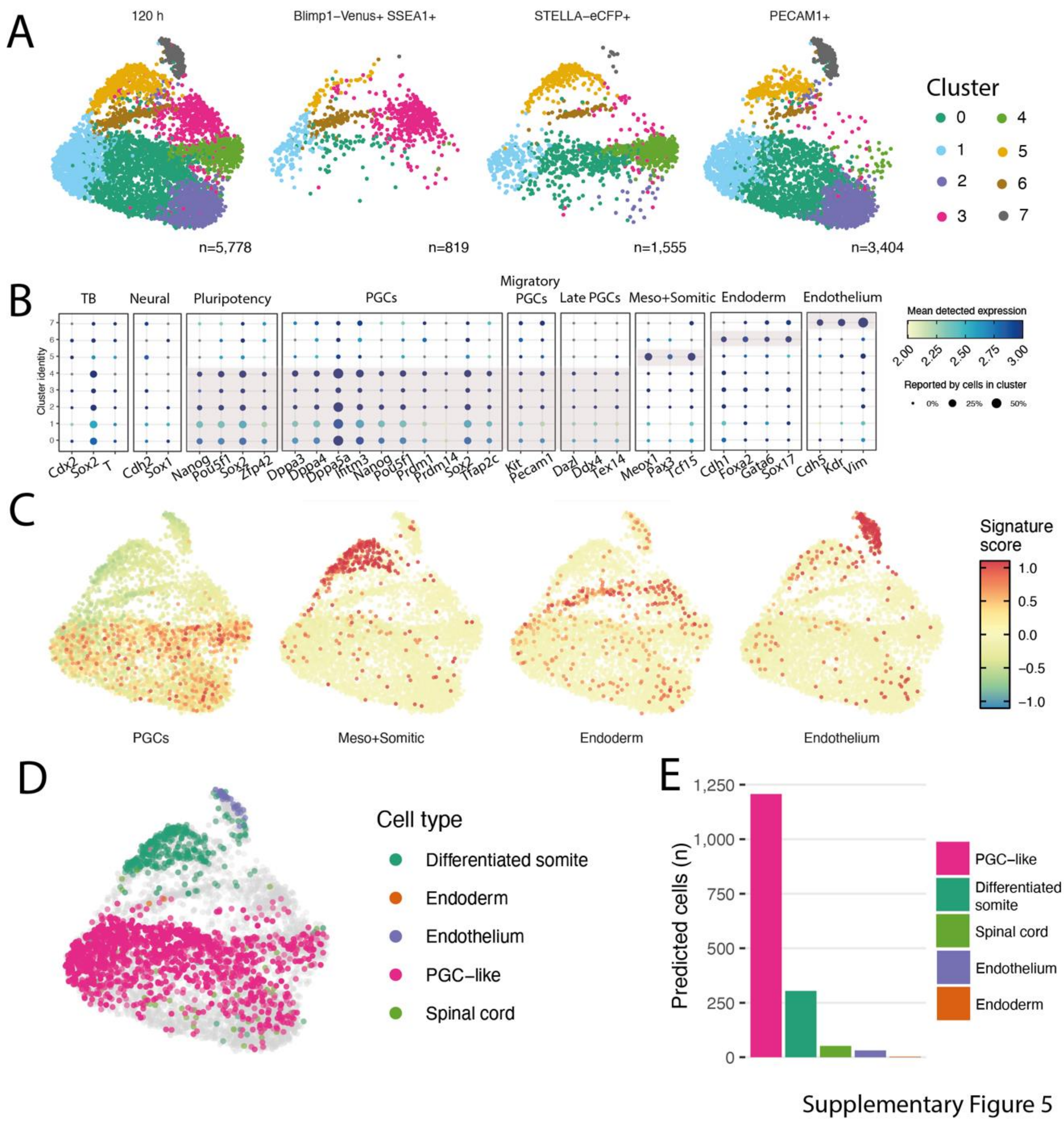

Supplementary Figure 5

Fig. S5. Analysis of single-cell transcriptomics. (A) UMAP of Gld-PGCLCs from all 3 sorted populations, showing 8 distinct clusters according to set parameters (see Methods). **(B)** Gene expression within clusters, grouped by biological categories. Shading indicates the potential identity of clusters: cluster 0-4 as PGC-like, cluster 5 as Meso+Somitic-like, cluster 6 as Endoderm-like and cluster 7 as endothelium-like. **(C)** Module Score information for selected gene signatures across the UMAP representation. **(D)** Cell label transfer from published gastruloid dataset[1], showing general concordance with gene expression annotation. **(E)** Quantification of cells assigned to identities by label transfer with the van den Brink dataset.

Development • Supplementary information

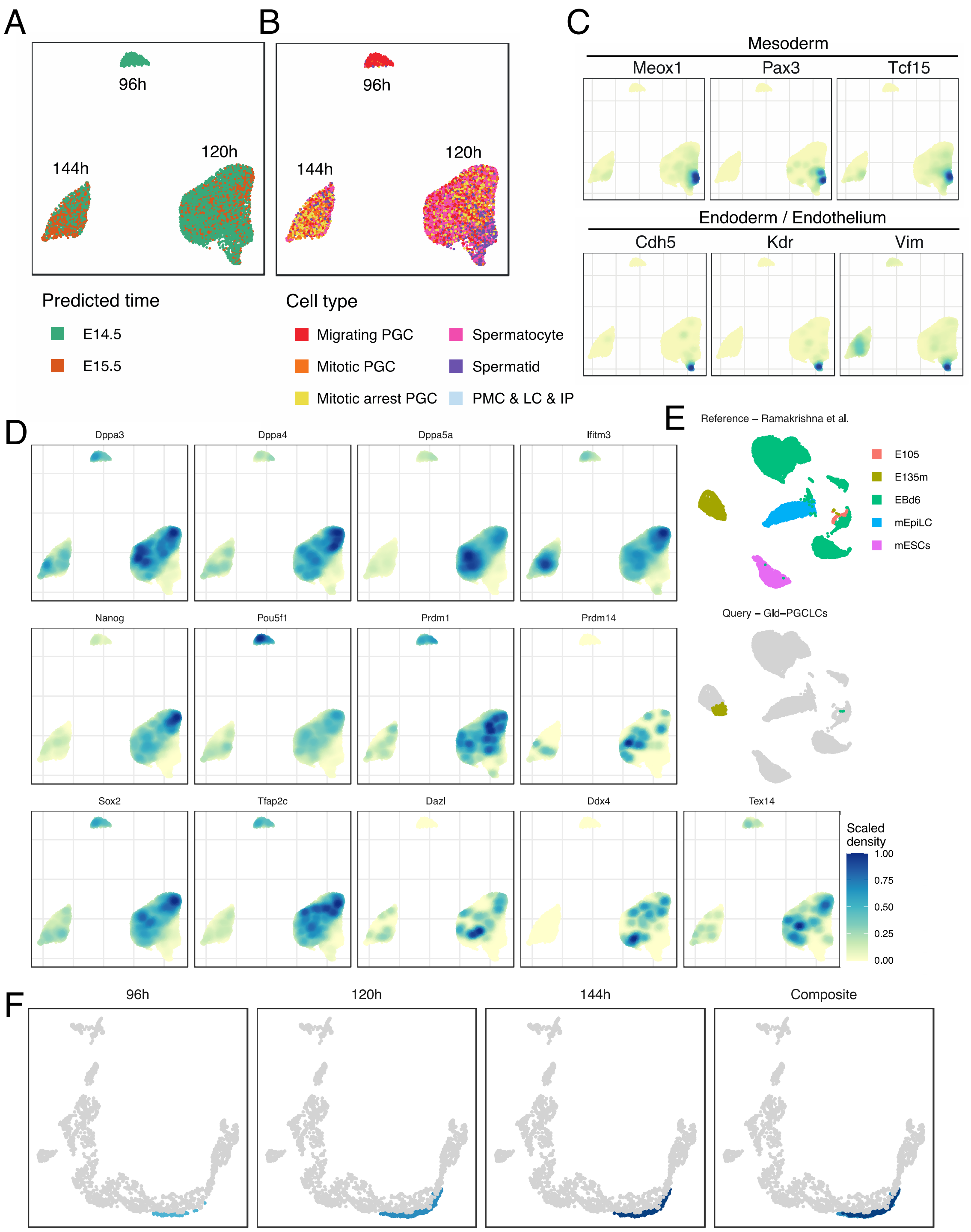

Supplementary Figure 6

Fig. S6. Time course single-cell RNAseq of Gld-PGCLCs. (A) Gld-PGCLCs sorted from gastruloids at 96, 120 and 144h post aggregation and coloured by embryonic timepoint labels transferred from *in vivo* Embryonic PGC data subset from Zhao and colleagues (see Methods for details). **(B)** Gld-PGCLCs from all sampled gastruloid timepoints, with *in vivo* Embryonic cell type labels transferred. **(C)** Mesodermal and endodermal marker gene expression of time course Gld-PGCLC dataset. Colourbar as in panel (D). **(D)** PGC marker gene expression of time course Gld-PGCLC dataset visualised using Nebulosa (see Methods). **(E)** Integrated Gld-PGCLCs across all timepoints projected onto Ramakrishna and colleagues' dataset, showing reference dataset (top) and projected Gld-PGCLCs (bottom). **(F)** Projection of Gld-PGCLCs across all timepoints onto *in vivo* Embryonic PGC dataset. Reference map as shown in Figure 5.

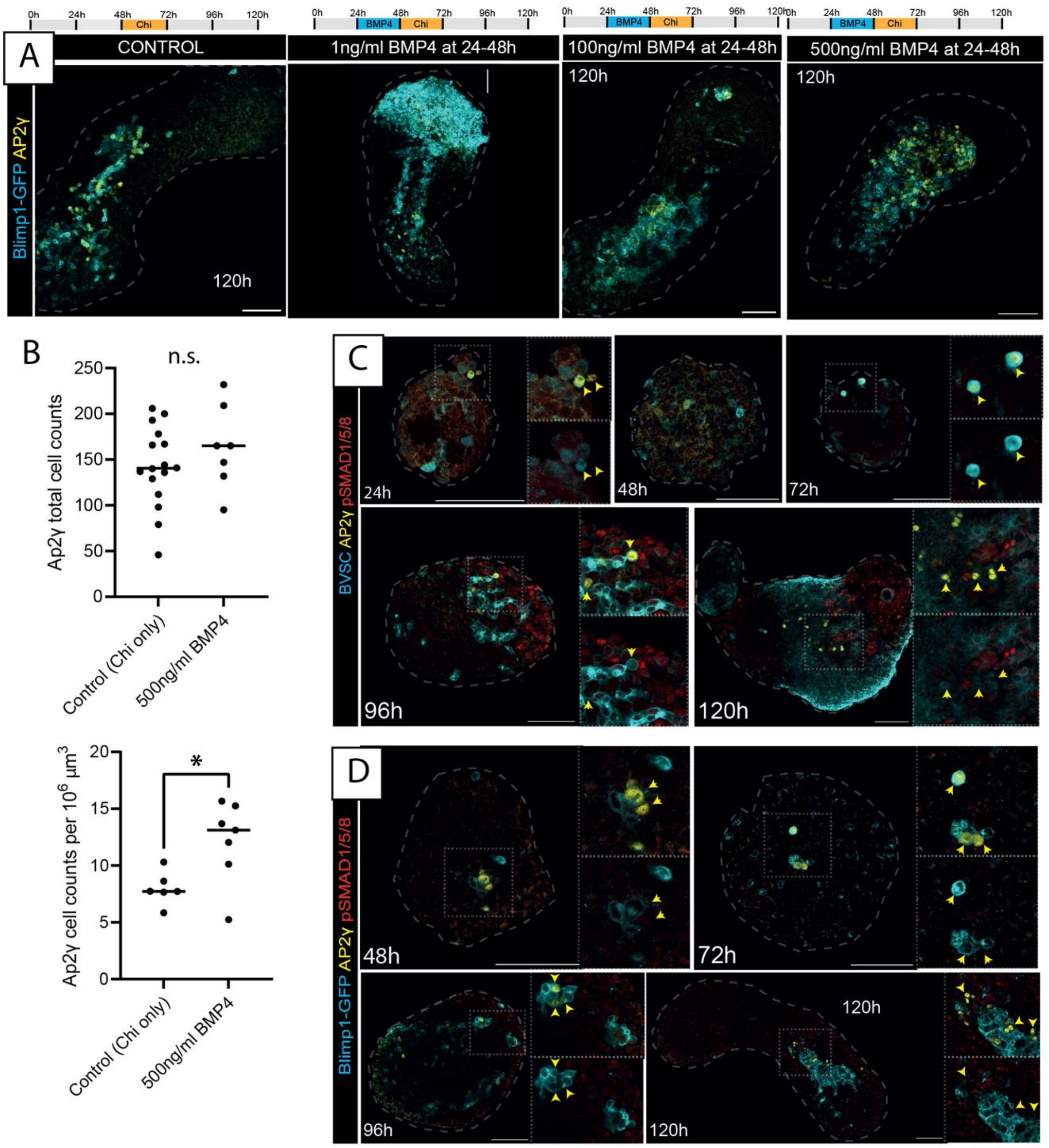

Supplementary Figure 7

Fig. S7. BMP signalling and Gld-PGCLCs. (A) Effect of BMP4 addition on Blimp1-GFP gastruloids at time indicated. **(B)** Quantification of AP2γ+ cell counts in Blimp1-GFP gastruloids at 120h. **(C-D)** Localisation of phospho-SMAD1/5/8 in BVSC **(C)** and Blimp1-GFP **(D)** gastruloids. Yellow arrowheads, Gld-PGCLCs. Insets, higher magnification images; Dashed line, morphological gastruloid outline from Hoechst staining; Dotted line, magnification region. Scale bars, 100 µm.

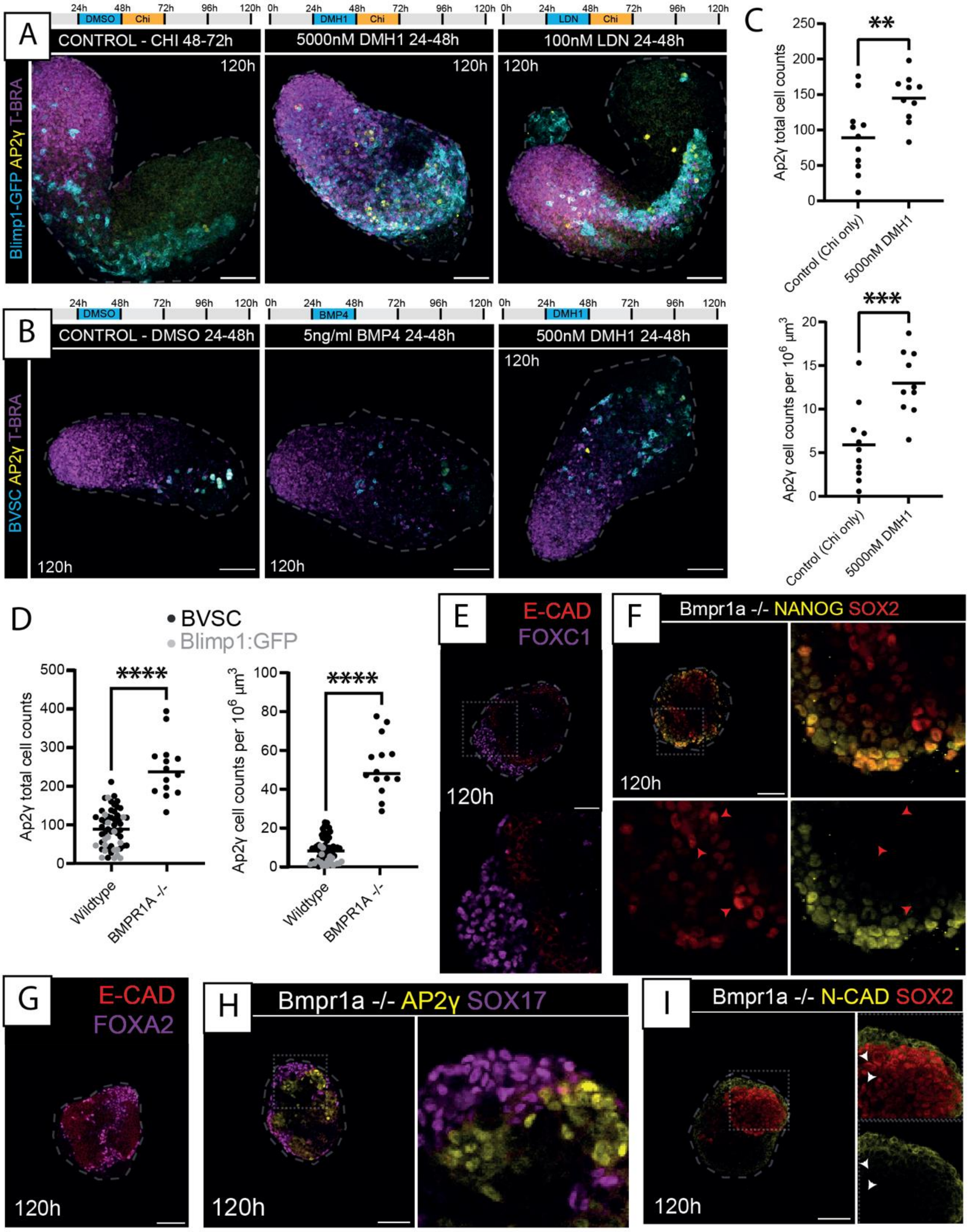

Supplementary Figure 8

Fig. S8. Effect of reduced BMP signalling on Gld-PGCLCs. (A) Inhibition of BMP signalling by DMH1 or LDN1 at times indicated. **(B)** Effect of BMP signalling modulation in the absence of Wnt signalling through Chi exposure. **(C)** Quantification of AP2γ+ cell counts in Blimp1-GFP gastruloids at 120h following DMH1 exposure. **(D)** Quantification of AP2γ+ cell counts in

Development • Supplementary information

BVSC and Blimp1-GFP gastruloids (Wildtype) or BMPR1A-/- at 120h. **(E-I)** BMPR1A-/- gastruloids show presence of markers of mesoderm, FOXC1, **(E),** neural, SOX2, **(F),** endoderm, FOXA2 and SOX17**, (G, H)** and ectoderm, N-Cadherin **(I).** Red arrowheads, SOX2+ NANOG- ectodermal cell examples. White arrowheads, SOX2+ N-CAD- Gld-PGCLC examples. Insets, higher magnification images; Dashed line, morphological gastruloid outline from Hoechst staining; Dotted line, magnification region. Scale bars, 100 µm.

Development • Supplementary information

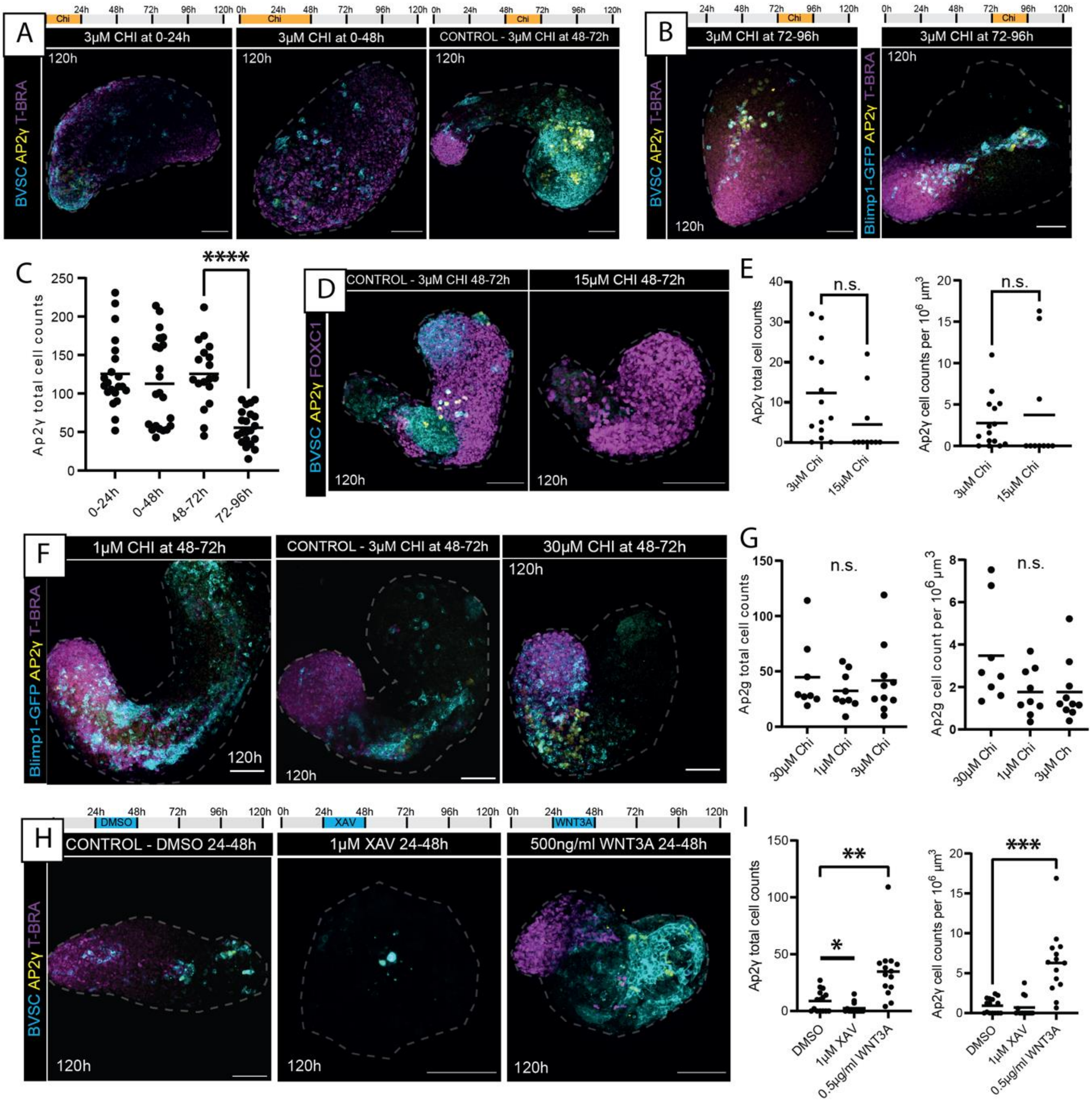

Supplementary Figure 9

Fig. S9. Wnt signalling and Gld-PGCLCs. (A-B) Maximum projection of gastruloids following shifts in Chi exposure, at timepoint and concentrations indicated. **(C)** Quantification of AP2γ+ cell counts in BVSC gastruloids at 120h following Chi exposure at timepoints indicated. **(D)** Maximum projection of BVSC gastruloids following increase in Chi concentration at timepoints indicated. **(E)** Quantification of AP2γ+ cell counts in BVSC gastruloids at 120h following Chi concentration modulation at 48-72h. **(F)** Maximum projection of Blimp1-GFP gastruloids following increase in Chi concentration at timepoints indicated. **(G)** Quantification of AP2γ+ cell counts in Blimp1-GFP gastruloids at 120h

Development • Supplementary information

following Chi concentration modulation at 48-72h. **(H)** Maximum projection of BVSC gastruloids following Wnt signalling modulation by ligand application, Wnt3a, or inhibitor exposure, XAV, in the absence of Chi pulse. **(I)** Quantification of AP2γ+ cell counts in BVSC gastruloids at 120h following Wnt signalling modulation, at timepoint and concentrations indicated. Dashed line, morphological gastruloid outline from Hoechst staining; Dotted line, magnification region. Scale bars, 100 µm.

Development • Supplementary information

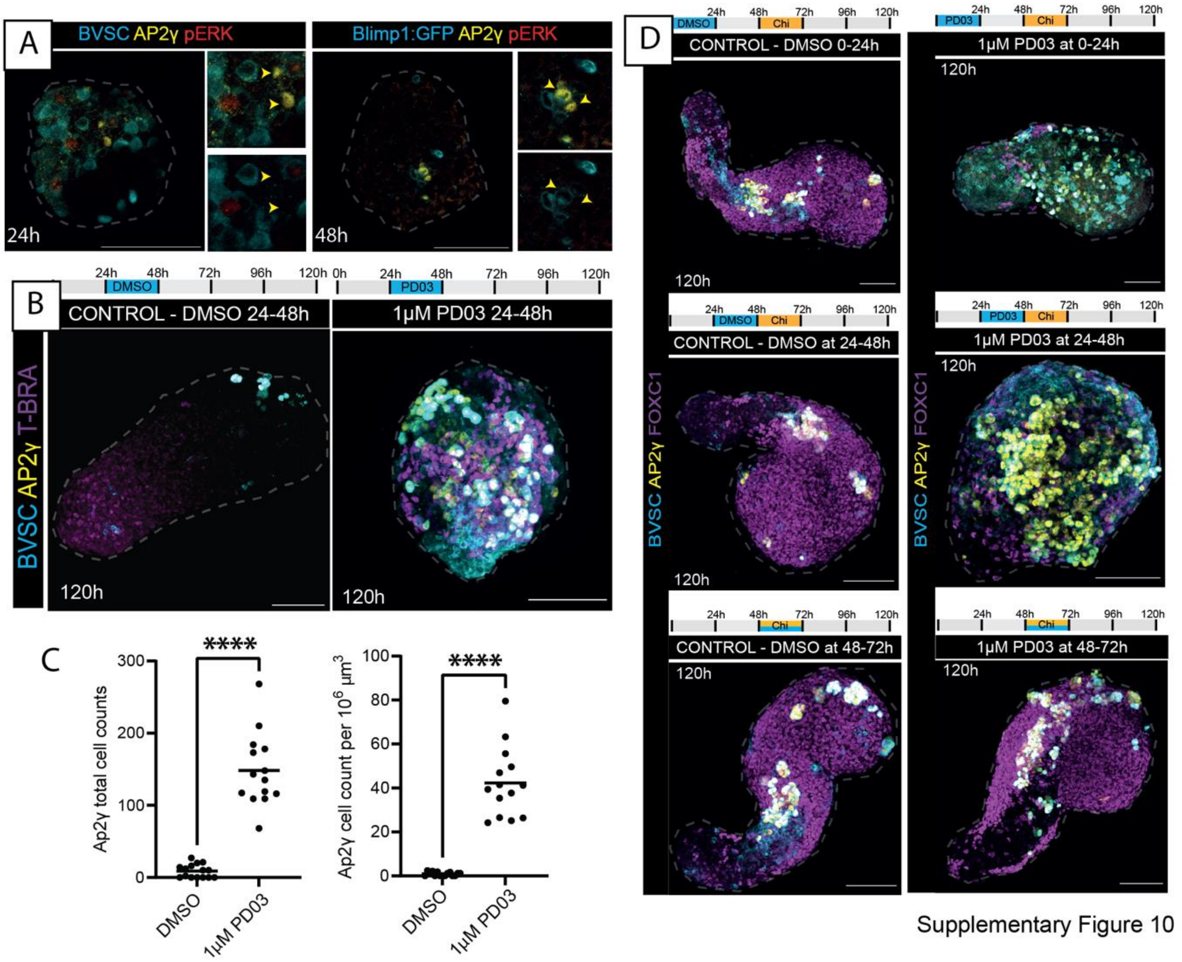

Supplementary Figure 10

Fig. S10. FGF signalling and Gld-PGCLCs. (A) Z slice of phospho-ERK in BVSC (left) and Blimp1-GFP (right) gastruloids at onset of Gld-PGCLC specification. Yellow arrowheads, AP2γ+ pERK- cells. **(B)** Maximum projection of BVSC gastruloids with inhibition of FGF signalling by exposure to PD03 at time and concentration indicated, in the absence of Chi pulse. **(C)** Quantification of AP2γ+ cell counts in BVSC gastruloids at 120h following PD03 exposure at 24-48h, in the absence of Chi pulse. **(D)** Maximum projection of BVSC gastruloids following FGF signalling modulation by PD03 at timepoints indicated. Insets, higher magnification images; Dashed line, morphological gastruloid outline from Hoechst staining; Dotted line, magnification region. Scale bars, 100 µm.

Table S1. Table of gastruloid lengths and volumes, from BVSC and Blimp1-GFP gastruloids at 120h and 144h.

120h

Gastruloid measurements	Gastruloid type: BVSC	Gastruloid type: Blimp1-GFP
Mean Length (µm) (± SD)	607.00 ± 122.20	933.07 ± 106.00
Mean volume (μm^3) (± SD)	$0.66 \times 10^7 \pm 2.62 \times 10^6$	$2.17 \times 10^7 \pm 5.90 \times 10^6$
Number of samples	32	16

144h

Gastruloid measurements	Gastruloid type: BVSC	Gastruloid type: Blimp1-GFP
Mean Length (µm) (± SD)	301.9 ± 45.53	1014 ± 205.5
Mean volume (μm^3) (± SD)	$0.33 \times 10^7 \pm 0.24 \times 10^6$	$1.86 \times 10^7 \pm 5.82 \times 10^6$
Number of samples	24	18

Table S2. Table of number of cells per Gld-PGCLC cluster in 144h gastruloids.
Gast, Gastruloid replicate.

	Gast 1					Gast 2					Gast 3	Gast 4			Gast 5			Gast 6	Gast 7				
Cluster no.	1	2	3	4	5	6	7	8	9	10	11	12	13	14	15	16	17	18	19	20	21	22	23
AP2γ + PGC marker	0	2	1	4	3	6	4	7	6	0	0	4	3	8	4	2	5	7	5	6	5	6	8
AP2γ + Blimp1	5	6	6	1	3	0	0	5	0	0	12	0	0	0	0	0	0	0	0	0	0	0	0
PGC marker + Blimp1	0	0	0	0	0	0	0	0	0	0	0	0	0	0	0	0	0	0	0	0	2	0	0
AP2γ + PGC marker + Blimp1	0	6	3	0	0	3	0	0	8	0	1	4	13	5	16	1	0	2	2	0	3	0	4
Total	5	14	10	5	6	9	4	12	14	0	13	8	16	13	10	13	5	9	7	6	10	6	12
Total cells in cluster	9	14	11	7	10	9	6	15	14	10	13	8	16	13	10	13	5	9	13	6	10	7	12

Table S3. Table of number and percentage of cells co-expressing AP2g with additional PGC markers, including DAZL, 5hmC and H3K27me3.

	Mean total Ap2γ cell count (± SD)	Mean total DAZL cell count (± SD)	Mean total co-expressed cell count (± SD)	Mean % co-expressed in Ap2γ cells	Mean % co-expressed in DAZL cells
BVSC 144h (n = 8)	225.9 ± 112.2	26.1 ± 10.2	23.3 ± 10.3	10%	89%
Blimp1-GFP 144h (n = 7)	102.1 ± 63.2	71.9 ± 48.2	49.6 ± 38.1	49%	69%
Combined 144h (n = 15)	168.13 ± 110.0	47.5 ± 40.1	35.5 ± 29.3	21%	75%

	Mean total Ap2γ cell count (± SD)	Mean total 5hmC cell count (± SD)	Mean total co-expressed cell count (± SD)	Mean % co-expressed in Ap2γ cells	Mean % co-expressed in 5hmC cells
BVSC 144h (n = 6)	133.0 ± 47.1	80.5 ± 30.5	64.2 ± 30.5	48%	80%
Blimp1-GFP 144h (n = 3)	41.3 ± 23.8	23.7 ± 19.7	11.0 ± 3.5	27%	46%
Combined 144h (n = 9)	102.4 ± 60.2	61.6 ± 38.6	46.4 ± 35.9	45%	75%

	Mean total Ap2γ cell count (± SD)	Mean total H3K27me3 cell count (± SD)	Mean total co-expressed cell count (± SD)	Mean % co-expressed in Ap2γ cells	Mean % co-expressed in H3K27me3 cells
BVSC 144h (n = 8)	146.1 ± 61.9	80.1 ± 16.6	50.1 ± 19.1	34%	63%
Blimp1-GFP 144h (n = 2)	63.5 ± 2.1	157.5 ± 26.2	24.0 ± 8.5	38%	15%
Combined 144h (n = 10)	129.6 ± 64.8	95.6 ± 36.8	44.9 ± 20.3	35%	47%

Development • Supplementary information

Table S4. Guide RNA sequences for CRISPR/Cas9 targeting and validation.

Name	Sequence (5'-3')
gRNA1	aaacTCAAATGTCGGGGTAGTTGC
gRNA2	aaacGATGCGGGATACGCCAGTGAc
hU6-F	GAGGGCCTATTTCCCATGATT
P1 (Fwd; wt Sox17)	GCTTTACGAGTTCCTCTGGGC
P2 (Rev; 3' UTR Exon 5)	GGCAAATTTTGTGGGAAGTGGG
P3 (Rev; eGFP)	CGTTGGGGTCTTTGCTCAGG
P4 (Rev; wt Sox17)	CCATGTGCGGAGACATCAGC

Table S5. Signalling modulators

Name	Supplier
Bone morphogenetic protein 4 (BMP4)	R&D Systems, 314-BP
CHIR99021 (Chiron or CHI)	Cambridge Stem Cell Institute
Dorsomorphin Homologue 1 (DMH1)	MedChem Express, HY-12273
LDN 193189 dihydrochloride (LDN)	Tocris Biosciences, 6053
PD0325901 (PD03)	Cambridge Stem Cell Institute
Wnt3a (Wnt family Member 3a) protein	Abcam, ab81484
XAV939 (XAV)	Selleck Chemicals, S1180

Table S6. Primary antibodies

Antibody target	Host species	Supplier	Catalogue number	Dilution
5hmC	Rabbit	Abcam	ab214728	1 in 100
AP2-gamma	Mouse	Santa Cruz	sc-53162	1 in 100
Brachyury	Rabbit	Abcam	ab209665	1 in 100
DAZL	Rabbit	Abcam	ab215718	1 in 200
E-cadherin	Mouse	Abcam	ab76055	1 in 100
E-cadherin	Rat	Takara	M108	1 in 100
EpCAM	Rabbit	Abcam	ab221552	1 in 100
FoxA2	Rabbit	Abcam	ab108422	1 in 100
FoxC1	Rabbit	Abcam	ab223850	1 in 200
GATA4	Rabbit	Abcam	ab84593	1 in 200
GCNA1 (Tra98)	Rat	Abcam	ab82527	1 in 200
GFP	Chicken	Abcam	ab13970	1 in 2000
Histone H3K27me3	Rabbit	Abcam	ab192985	1 in 100
Nanog	Rat	ThermoFisher Scientific	14-5761-80	1 in 250
Nanog	Rabbit	Abcam	ab214549	1 in 100
N-cadherin	Mouse	BD Biosciences	610921	1 in 200
Oct4	Rabbit	Abcam	ab200834	1 in 200
PECAM1 (CD31)	Rat	BD Biosciences	557355	1 in 200
PhosphoERK	Rabbit	Cell Signalling Technology	4370	1 in 100
PhosphoSMAD1/5/8	Rabbit	Cell Signalling Technology	13820	1 in 100
Stella (DPPA3)	Goat	R&D Systems	AF2566	1 in 50
Sox2	Rabbit	Abcam	ab92494	1 in 200
Sox17	Rabbit	Abcam	ab224637	1 in 100
CDX2	Rabbit	Abcam	ab76541	1 in 200

Table S7. Secondary antibodies and primary conjugate

Antibody target	Antibody species/type	Supplier	Catalogue number	Dye
Chicken IgY	Goat	Abcam	ab150173	Alexa 488
Mouse IgG	Donkey	ThermoFisher	A10037	Alexa 568
Mouse IgG	Goat	ThermoFisher	A21236	Alexa 647
Rabbit IgG	Donkey	ThermoFisher	A21206	Alexa 488
Rabbit IgG	Donkey	ThermoFisher	A10042	Alexa 568
Rabbit IgG	Donkey	ThermoFisher	A31573	Alexa 647
Rat IgG	Donkey	ThermoFisher	A21208	Alexa 488
Rat IgG	Donkey	Abcam	ab150153	Alexa 647
PECAM1(CD31)	Rat	BD Biosciences	553373	Phycoerythrin (PE)

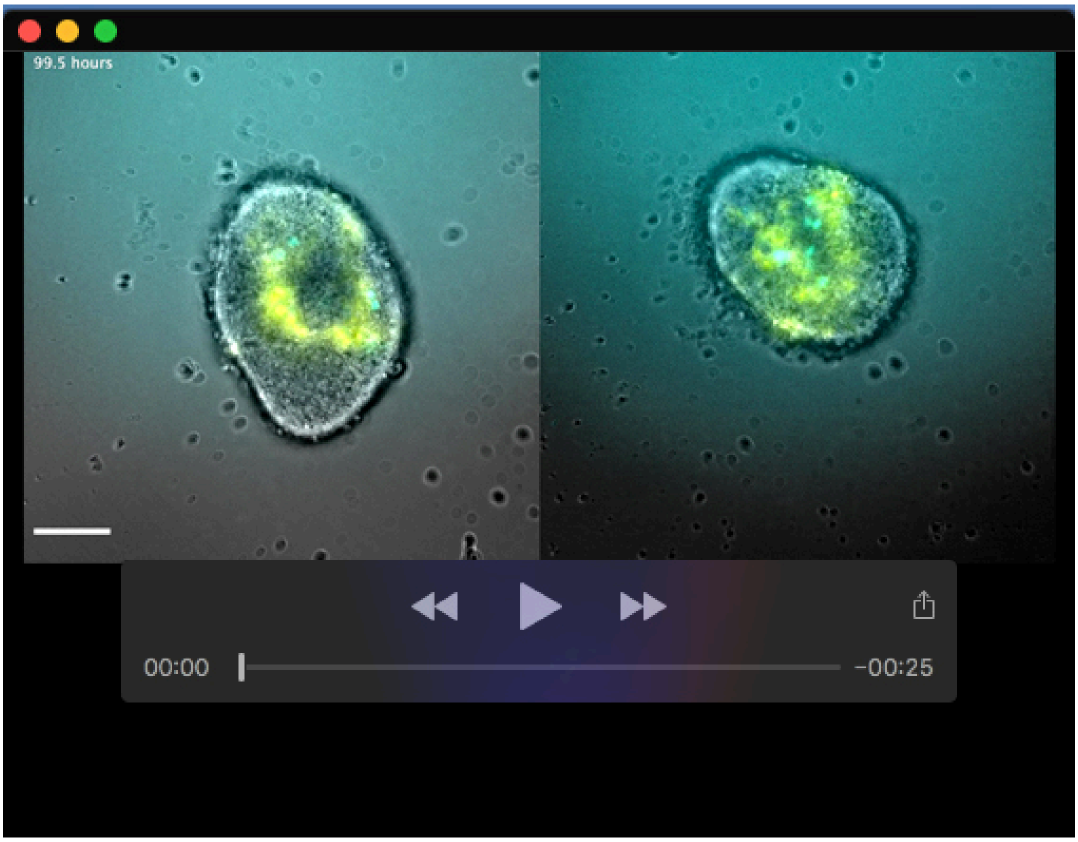

Movie 1. Widefield imaging of two BVSC gastruloids from 98h after aggregation, imaged at 20 minute intervals. Cyan, Blimp1-GFP; Yellow, Stella:eCFP. Scale bar = 100 µm.

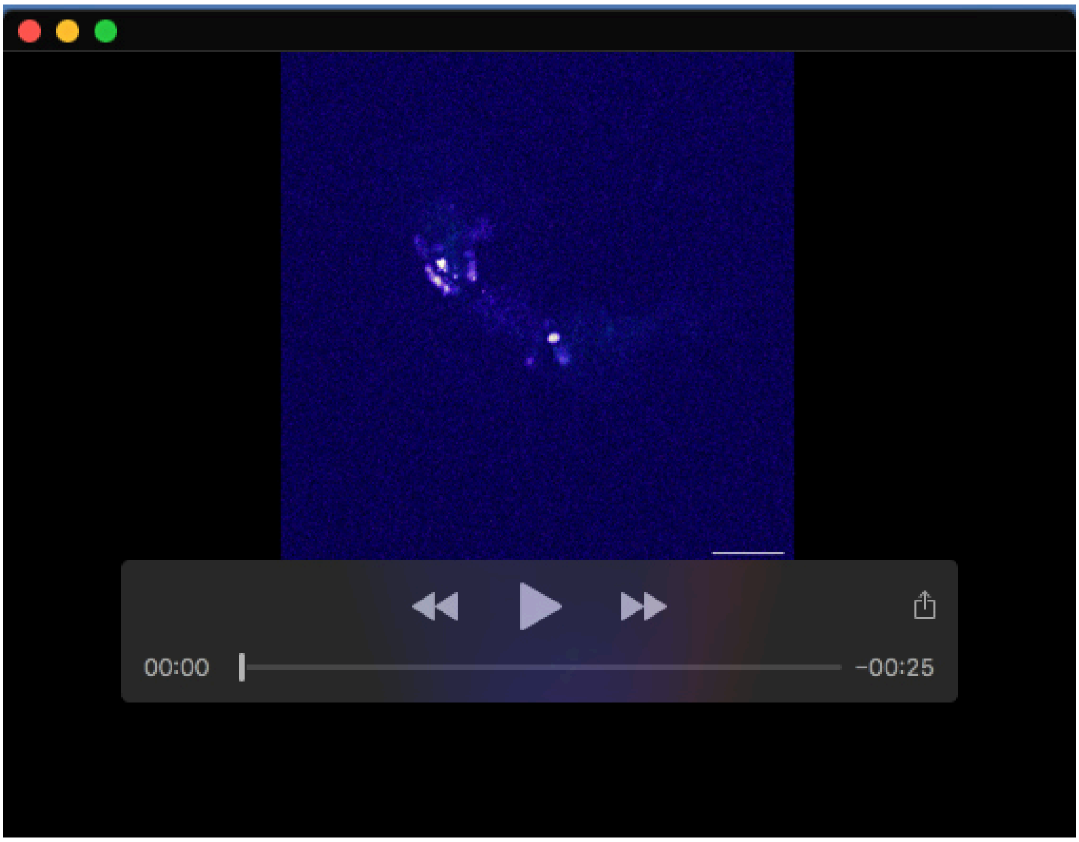

Movie 2. Multiphoton live-imaging movie of a BVSC gastruloid from 118h after aggregation, imaged at 30 minute intervals. Cyan, Blimp1-GFP; Yellow, Stella:eCFP. Scale bar = 100 µm.

Supplementary reference

1. van den Brink, S. C. *et al.* (2020) Single-cell and spatial transcriptomics reveal somitogenesis in gastruloids. *Nature* 582, 405-9 https://doi.org/10.1038/s41586-020-2024-3.

Development • Supplementary information
